# Extensive differential DNA methylation between tuberculosis skin test positive and skin test negative cattle

**DOI:** 10.1186/s12864-024-10574-x

**Published:** 2024-08-06

**Authors:** Sajad A. Bhat, Alia Parveen, Eamonn Gormley, Kieran G. Meade

**Affiliations:** 1 UCD School of Agriculture and Food Science, University College Dublin, Belfield, Dublin, D04 V1W8 Ireland; 2 UCD School of Veterinary Medicine, University College Dublin, Belfield, Dublin, D04 V1W8 Ireland; 3 UCD Conway Institute of Biomolecular and Biomedical Research, University College Dublin, Belfield, Dublin, D04 V1W8 Ireland; 4 UCD Institute of Food and Health, University College Dublin, Belfield, Dublin, C15 PW93 Ireland

**Keywords:** Methylation, Bovine tuberculosis, Tuberculin skin test, Interferon gamma, Asymmetric methylation, Diagnostics

## Abstract

Bovine tuberculosis (bTB), caused by *Mycobacterium bovis* (*M. bovis*), represents a significant problem for the agriculture industry as well as posing a risk for human health. Current diagnostic tests for bTB target the cell-mediated immune (CMI) response to infection with *M. bovis*, primarily through screening of animals with the tuberculin skin test. Epigenetic modifications have been shown to alter the course of the immune response and differentially methylated regions (DMRs) might also influence the outcome of the skin test in cattle. Whole Genome Bisulphite Sequencing (WGBS) was used to profile DNA methylation levels from peripheral blood of a group of cattle identified as test positive for *M. bovis* (positive for the single intradermal comparative tuberculin test (SICTT) and/or the interferon-γ release assay compared to a test negative control group [*n* = 8/group, total of 16 WGBS libraries]. Although global methylation profiles were similar for both groups across the genome, 223 DMRs and 159 Differentially Promoter Methylated Genes (DPMGs) were identified between groups with an excess of hypermethylated sites in SICTT positive cattle (threshold > 15% differential methylation). Genes located within these DMRs included the Interleukin 1 receptor (*IL1R1*) and MHC related genes (*BOLA* and *BOLA-DQB*). KEGG pathway analysis identified enrichment of genes involved in Calcium and MAPK signalling, as well as metabolism pathways. Analysis of DMRs in a subset of SICTT negative cattle that were IFN-γ positive showed differential methylation of genes including Interleukin 10 Receptor, alpha (*IL10RA*), Interleukin 17 F (*IL17F*) and host defence peptides (*DEFB* and *BDEF109*). This study has identified a number of immune gene loci at which differential methylation is associated with SICTT test results and the degree of methylation could influence effective host immune responses.

**Supplementary Information:**

The online version contains supplementary material available at 10.1186/s12864-024-10574-x.

## Background

Infection by *Mycobacterium bovis* (*M. bovis*) continues to cause significant animal health issues globally with reservoirs of infection in both livestock and wildlife populations [1]. In addition to the significant costs associated with control and eradication programmes, *M. bovis* is a zoonotic pathogen and therefore has consequential trade and human health impacts [2]. Although the overall proportion of cattle herds testing positive for bTB across the EU remains low, certain countries are experiencing increasing incidences in recent years. As a result, intensive eradication efforts are in place in countries across the EU, including Ireland [3].

The current eradication programme for bTB in Ireland is underpinned by annual screening of cattle over six weeks of age with the Single Intradermal Comparative Tuberculin Test (SICTT), an in vivo skin test that measures the activation of a Delayed Type Hypersensitivity (DTH) reaction within 72 h after the administration of both bovine and avian mycobacterial tuberculin antigens (*M. bovis/M. avium* purified protein derivative – PPDb/PPDa) [4]. PPDa is used to control for the background sensitisation of animals with cross-reactive environmental mycobacteria and is applied to increase test specificity. A positive standard ‘reactor’ animal is disclosed when the DTH reaction of the response to PPDb is at least 4 mm (B-A > 4 mm) greater than the response to PPDa. More severe interpretations (e.g., B-A > 2 mm) can help to increase the sensitivity of the SICTT. In herds where bTB reactor animals are identified, the interferon- gamma assay (IFN-γ) is often used as an ancillary test, interpreted in parallel with the SICTT to improve the sensitivity of diagnostic testing. The principle of the IFN-γ assay is to detect and quantify release of the IFN-γ cytokine when heparinised whole blood is incubated with PPDb and PPDa [5]. All reactor animals are euthanised soon after disclosure and subjected to post-mortem examination at the abattoir for the presence of lesions consistent with *M. bovis* infection. However, lesion detection in reactor animals is relatively low: several studies have found that 50–80% of reactor animals had no visible lesions [6] and in a study carried out in Northern Ireland, only 43% of reactors had visible lesions detected at slaughter [7]. Nevertheless, the failure to detect visible lesions at post-mortem does not necessarily indicate absence of infection. The SICTT has a median specificity approaching 100% based on studies of bovine tuberculosis (bTB)-free populations from several countries [5, 6, 8, 9]. Therefore, when multiple SICTT reactors are identified in an exposed herd by the SICTT and / or the IFN-γ test, the probability that they are false-positive reactors is very low. They are deemed to be infected with *M. bovis*, even in the absence of confirmation by pathological examination. Likewise, in low-risk of infection cohorts, two negative tests (SICTT and the IFN-γ test) in any animal indicate a low probability of bTB infection. Animals that are positive in the IFN-γ test are also considered at higher risk of infection than IFN-γ test negatives [10–12].

In an effort to improve on the performance characteristics of current available diagnostic tests, the advent of new technologies, including transcriptomics, have also served to increase our understanding of the immune response in bTB infected cattle [13, 14]. These studies have revealed a complex array of gene expression patterns that govern the specific responses to infection with *M. bovis* [15, 16]. An emerging area of research has also shown that these responses are underpinned by epigenetic processes that modify chromatin structure to facilitate the coordinated transcription of genes involved in the specific immune responses to infection [17, 18].

Epigenetics refers to chemical modifications to DNA and proteins which control access by the transcriptional machinery to the underlying genetic sequence and is relevant to all phenotypic traits in verterbrates [19, 20]. As each step in the immune response to bTB relies on gene activation prior to the translation of effector proteins, epigenetic modifications are involved in regulation of the IFN-γ and ultimately DTH responses [21]. It has also been shown that infectious organisms, including mycobacteria have evolved means to manipulate the epigenome of their host to facilitate their survival [22, 23]. One major epigenetic mechanism is methylation of DNA through the addition of a methyl group to the cytosine residue of DNA, often in the context of a CpG island [24]. However, other forms of asymmetrical methylation have also been identified in mammals, where methylation occurs to the cytosine in an alternative context - namely CHG or CHH (where H correspond to A, T or C) [25, 26]. The effects of methylation on the immune response will depend on its location: in the promoter region of some genes, for example, methylation can repress the binding of transcription factors potentially downregulating gene expression. Studies of cases with human tuberculosis (caused by *M. tuberculosis*) have identified specific loci at which hypermethylation results in a dampening of the immune response, including at the *IFNGR1* and *IL12BR2* genes [27]. Both IFN-γ and IL-12 cytokines are associated with the development of protective immunity to mycobacteria [28] and therefore hypermethylation of these and/or other genes could impact on the outcome of infection as well as the performance of diagnostic tests based on host responses.

In this study, we set out to determine if cattle stratified by routine field interpretation of their SICTT and IFN-γ test responses exhibit differentially methylated immune genes and pathways which underpin their divergent immune responses profiles.

## Results & discussion

### Summary of whole genome bisulphite sequencing (WGBS) data

**Fig. 1:**
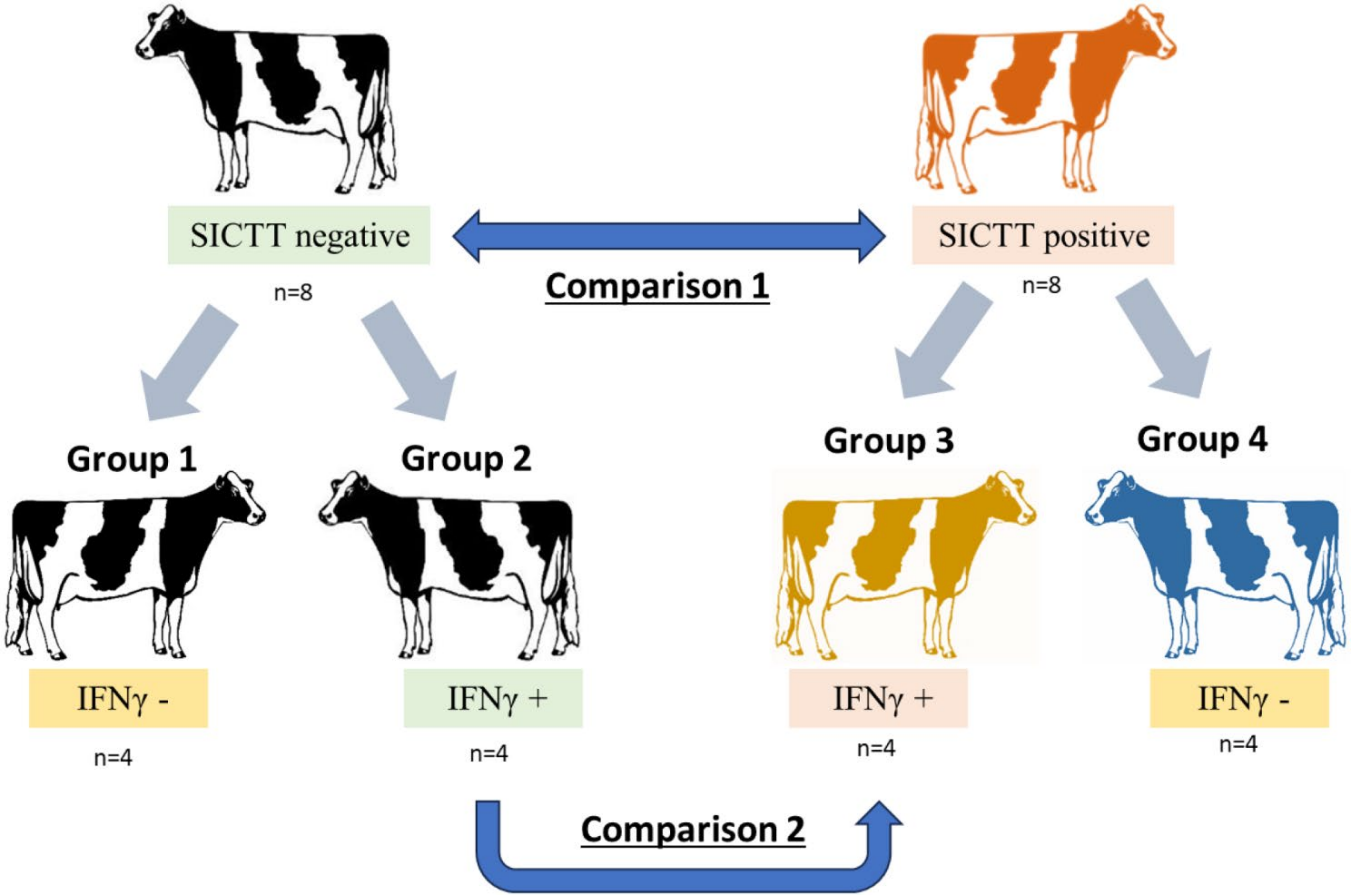
Experimental Design. Four cohorts of samples were identified based on diagnoses using the SICTT test results and IFN-γ assay results from naturally bTB + cattle or test negative controls. Eight SICTT negative and eight SICTT positive cattle were selected and further classified as either SICTT negative and IFN-γ negative (Group 1, *n* = 4); SICTT negative and IFN-γ positive (Group 2, *n* = 4); SICTT positive and IFN-γ positive (Group 3, *n* = 4); SICTT positive and IFN-γ negative (Group 4, *n* = 4)

Raw WGBS data from 16 whole blood samples representing bTB SICTT positive (*n* = 8) and negative (*n* = 8) cattle, each comprising of eight Holstein cows were assessed (Fig. 1 and Supplementary Table S1). After quality control, a total of 5.01 × 10^9^ clean reads were obtained with an average of 3.13 × 10^8^ (average 85.48 Gb per sample) for each sample, indicating a sequencing depth of 30X. GC content was 22.68% on average and the bisulphite conversion rate was 99.72% on average (Supplementary Table S2). On average, 78.88% of non-duplicated clean reads uniquely aligned to the bovine reference genome (ARS-UCD1.2) with an average duplication rate of 24%.

### DNA methylation levels and patterns across the bovine genome

On average, 3.38% of all cytosines in the genome were methylated and 64% of methylated cytosines occurred in a CG context (Supplementary Table S2). As expected, and previously documented in the literature, CG methylation representing the majority of methylation changes present, at 98% of methylated cytosines on average. Asymmetric methylation (CHG and CHH - where H is A, C, or T) accounted for a fraction of overall methylation changes, representing an average of 0.51% and 1.56% of methylated cytosines, respectively. No statistical differences in the proportion of CG, CHG or CHH methylated cytosines was detected between groups (P > 0.05). Across genomic features, clear, reproducible differences in methylation profiles was apparent across all samples with lowest CG methylation in the 5’UTR region of genes (20% on average) rising to maximal levels in exons (71%), introns (81%) and 3’ UTR regions (81%) (Supplementary Fig. S1A and S1B and Supplementary Table S2). The average levels of methylation across genomic features is remarkably similar to what has been reported in cattle recently, with 30% in the promoter and 5’ untranslated region (UTR), 68% in exons, 72% in introns, and 73% in the 3’ UTR [29]. Asymmetrical methylation patterns mirrored the CG profile in general but specific peaks of occurrences were apparent. At the gene level, the gene body accounted for the majority of CG methylation with peaks for CHG and CHH methylation immediately downstream of genes (Supplementary Fig. S2). Low levels of asymmetric methylation levels are also in agreement with what has previously been found in blood DNA samples from pigs [30]. The relevance of methylation in intronic and repeat regions of DNA have less clear relevance to disease processes, whereas methylation has a known role in promoter regions through the regulation of transcription factor binding and ultimately on gene expression [31]. Furthermore, methylation within gene exons is emerging as an important mechanism regulating alternative splicing [32] showing established functional relevance of methylation in these particular genomic regions.

## Comparison 1 - Whole genome methylation profile in SICTT positive and SICTT negative cattle reveals differential methylated regions and elevated asymmetrical methylation in SICTT positive cattle

**Fig. 2:**
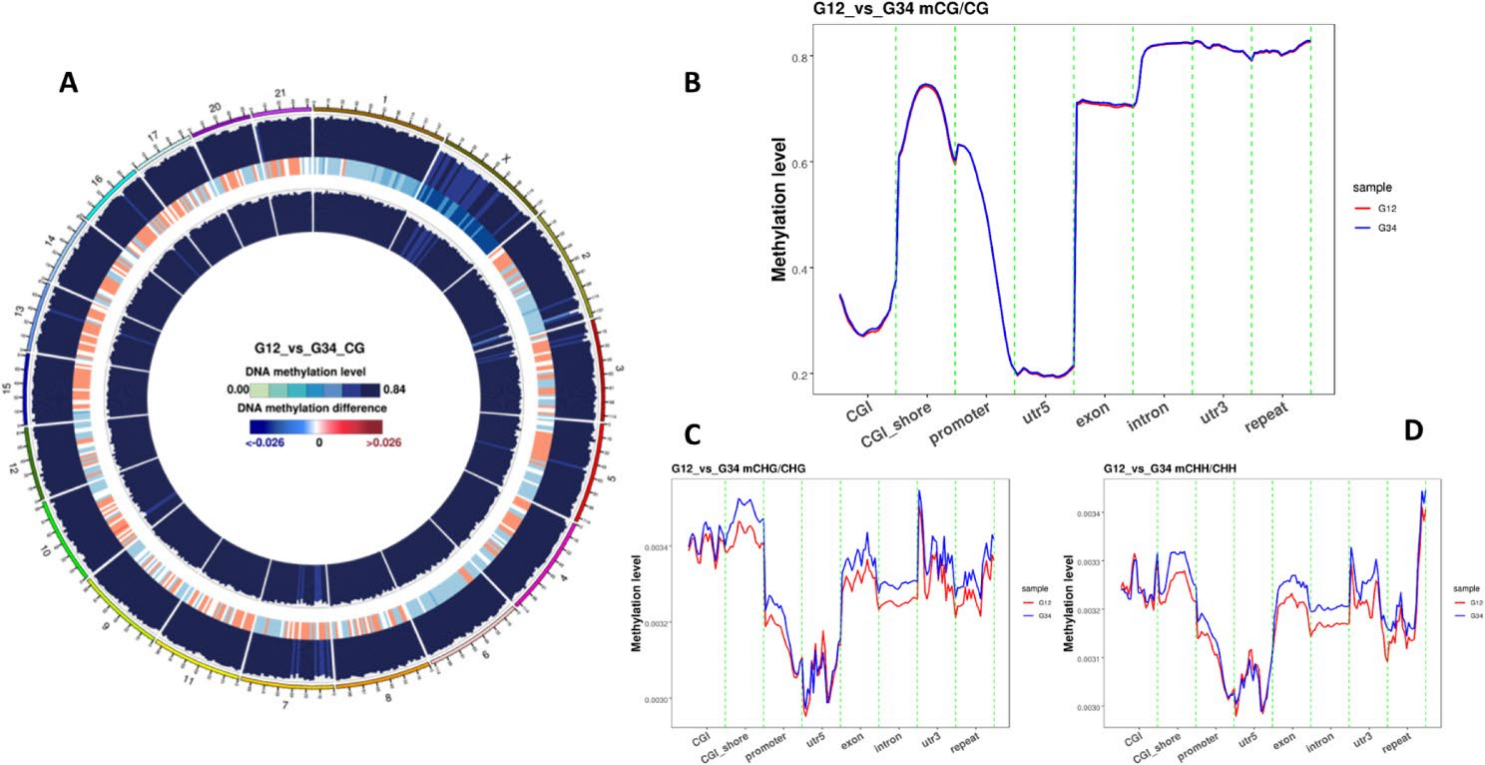
Differential methylation across the genome of bTB + cattle samples relative to test negative control samples. (A) Circos density plot of 5-methylcytosine density and difference across the genome. Chromosome numbers and scales are indicated on the periphery, a dark to light colour indicates a low to high level of methylation. Red indicates the greatest degree of methylation difference; (B) Differential CG methylation profile for bTB + cattle (shown in blue) and test negative controls (shown in red) across various genomic features; (C) Differential CHG methylation profile for bTB + cattle (shown in blue) and test negative controls (shown in red) across various genomic features; (D) Differential CHH methylation profile for bTB + cattle (shown in blue) and test negative controls (shown in red) across various genomic features. Both methylation types C and D are referred to as asymmetrical methylation

The methylation patterns of SICTT positive and negative cattle followed a similar pattern although differences were apparent and located widely across the genome (Fig. 2A). While overall levels of CG methylation were not statistically different between groups (Fig. 2B), the asymmetrical methylation patterns were quite distinct. Despite accounting for a minor fraction of the overall genomic methylation, both CHG and CHH methylation levels were elevated in the SICTT positive cattle DNA samples relative to the SICTT negative controls (Fig. 2C and D, respectively). Most obvious divergence in both CHG and CHH methylation levels was apparent in the CGI shores, gene promoter, exons and intronic regions. The role of asymmetric methylation is not fully understood [33], particularly in non-model organisms and while the levels reported in the bovine genome are low, the differential pattern in diseased cattle might be an important avenue for further investigation.

**Fig. 3:**
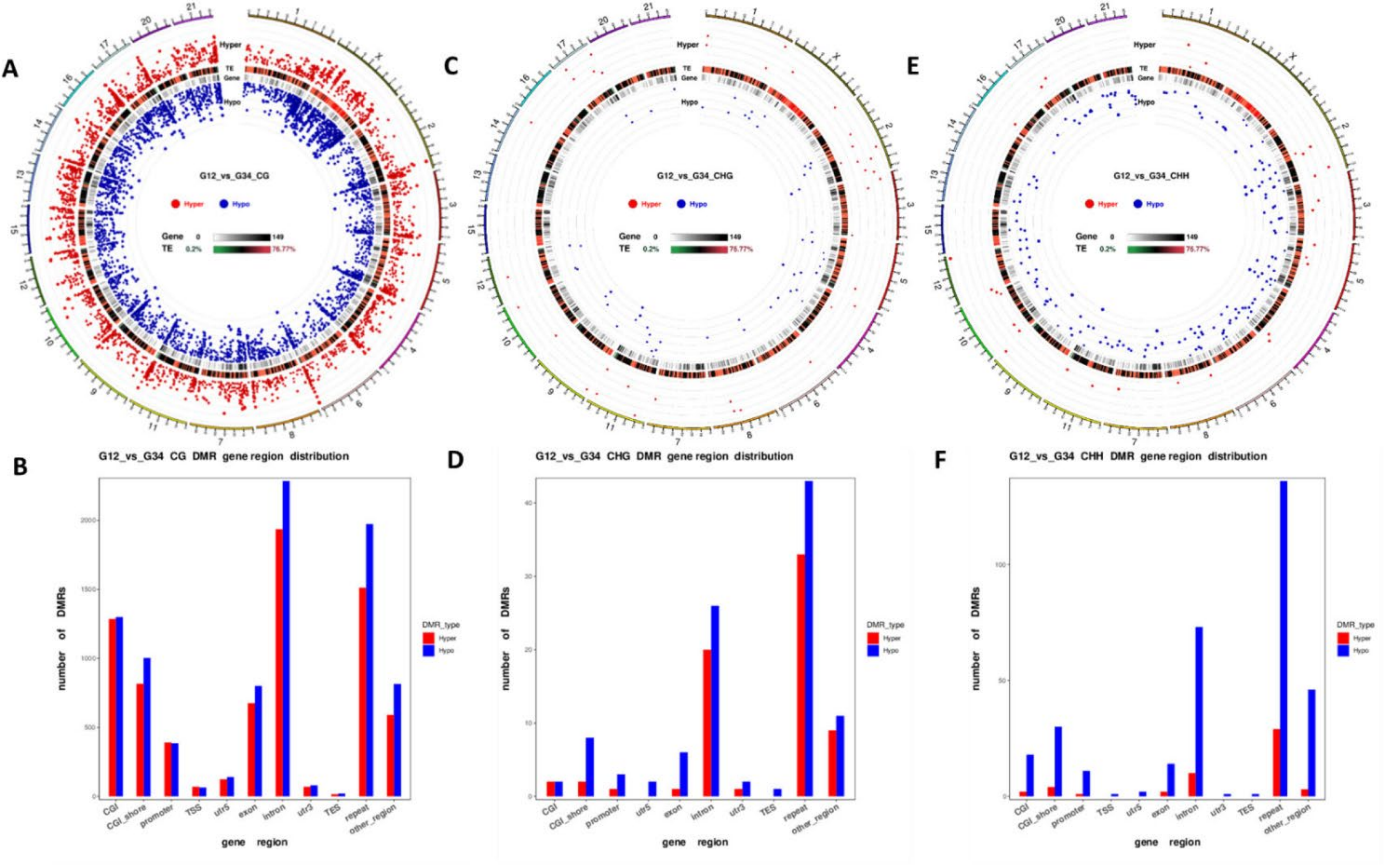
Ratio of hypermethylation to hypomethylation across the genome of bTB + cattle samples relative to test negative control samples. A circos density plot illustrates the degree of hypermethylation (shown in red) and hypomethylation (shown in blue) for (A) 5-methylcytosine (CG) methylation; (C) CHG methylation and (E) CHH methylation. Chromosome numbers and scales are indicated on the periphery. The ratio of hypermethylation to hypomethylation in each of the CG, CHG and CHH sites is shown for each genomic feature in (B), (D) and (F) respectively

Considering the direction of methylation differences across genomic features, hypermethylated and hypomethylated sites are spread across the genome (Fig. 3A). However, there is an overabundance of hypomethylated CG sites particularly in exons and introns (Fig. 3B). Although CHG and CHH methylation is a lot less abundant (Fig. 3C and E), an excess of hypomethylated sites is apparent in genomic regions including the promoter (Fig. 3D) and is most evident for CHH methylation patterns (Fig. 3F). From all identified DMRs, 8,410 mapped uniquely and were different between SICTT positive and negative controls across the genome (full list provided in Supplementary Table S3). Many of these DMRs represent extensive sequential runs of methylated cytosines spanning multiple genomic features (promoter, exon and introns) of the same gene, indicating extensive regulation of these genes by methylation (Supplementary Table S3). Various levels of stringency can be applied to increase the detection of DMRs with a potential functional impact including the areaStat and differential methylation values. The areaStat is the sum of the test statistics of all CpG sites within a DMR [34] and values in this study varied from − 1779 to 10,426 with a higher number associated with longer DMRs and are thought to indicate a more reliably detected DMR. However, various combinations of these parameters have been used in the literature to increase the stringency of DMR identification and we have used a similar approach to that previously adopted [35, 36] and focused on DMRs with a differential methylation level > 15% between groups for discussion, a level above which a biological relevance in a disease context is more likely.

A total of 1507 DMRs were identified in exons, which is reduced to 223, when the minimum 15% differential methylation cut off is applied (Table 1A). The range in differential methylation varied between − 0.49 and 0.46 and consecutive CG sites varied from a low of 4 to a maximum of 420 (median of 18 CpGs). In addition, a total of 833 DPMGs were identified, again reduced to 159 with > 15% differential methylation. Differential methylation levels in gene promoters were higher than in exons, varying from − 0.54 to 0.5 and the CpG sites were shorter than those found in exons, with a maximum of 119 CpGs in any DMR (median 12 sites). In both exons and gene promoters, the numbers of hypermethylated sites exceeded that of hypomethylated sites (124 and 90, compared to 99 and 69 respectively, threshold > 15%) in bTB + cattle compared to test negative controls (Table 1A).

**Fig. 4:**
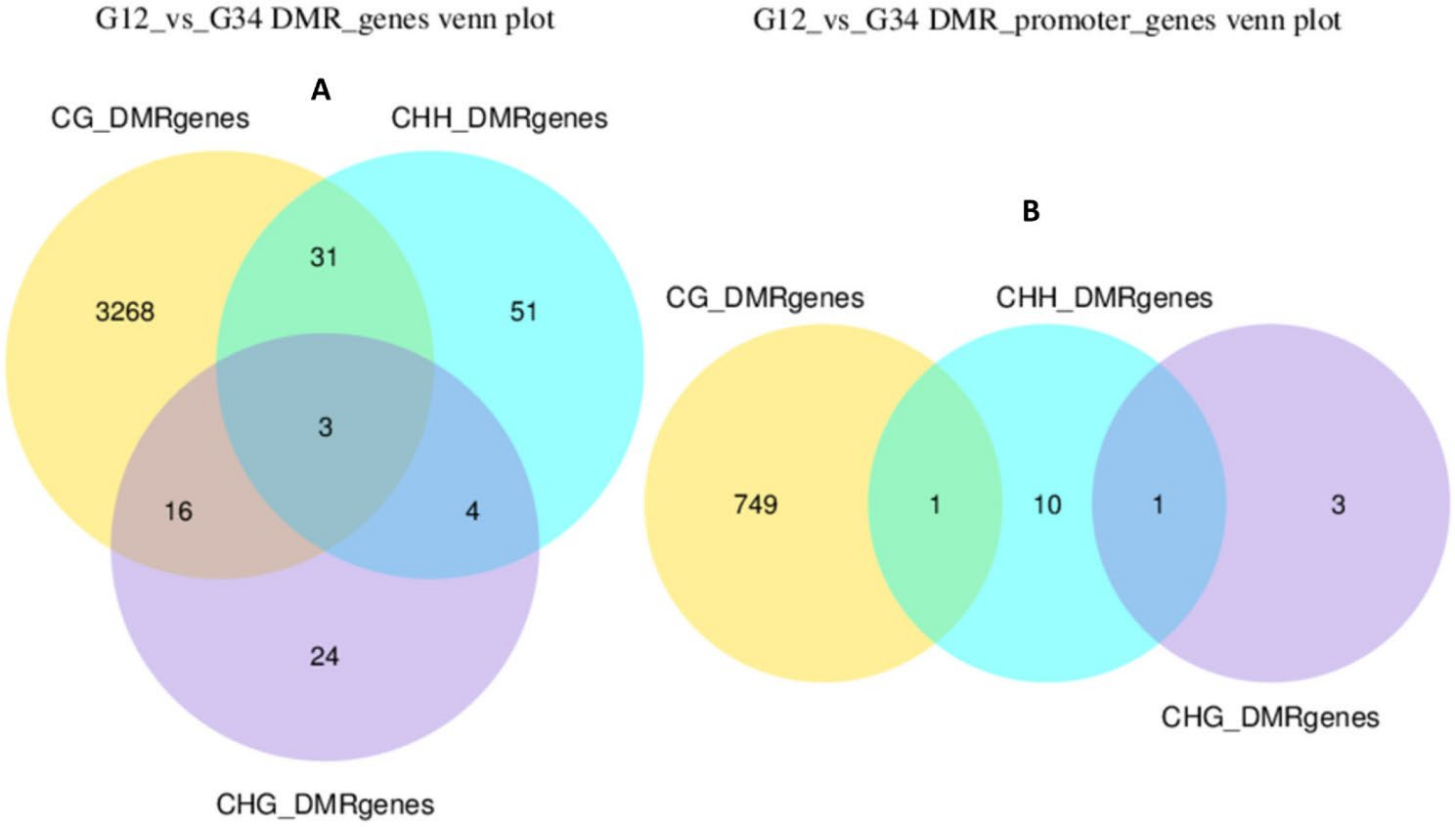
Numbers of genes identified as differentially methylated (CG, CHG or CHH) in (A) genes or (B) in the promoter region of genes of bTB + cattle samples relative to test negative control samples. The degree of overlap shows the numbers of genes with multiple forms of methylation. The identity of these genes is given in Supplementary Table S4

The number of genes identified within all DMRs is shown in Fig. 4A and B. The majority of these genes (3268 DMR genes and 749 DPMGs) are CG methylated and the top ranked differentially methylated DMR genes and DPMGs in bTB + cattle relative to test negative controls are shown in Table 1B and Table 1C. Amongst these hypermethylated genes is the Leukocyte Associated Immunoglobulin Like Receptor (*LAIR1*) gene, which is a regulator of hypersensitivity reactions [37], and shows 26% higher methylation in SICTT positive cattle samples. Immunoglobulin superfamily 6 (*IGSF6*), which is part of panel of genes proposed to differentiate between human TB patients at different stages of infection [38] shows 21% higher methylation. Immune responses to mycobacterial infection requires the presentation of antigen by innate immune cells via the major histocompatibility complex (MHC) [39] and 39% hypermethylation of the BoLa class II histocompatibility antigen (*BOLA-DQB*) gene may lead to sub-optimal CD4^+^ T cell activation (Table 1B). Mycobacterial peptides are also presented via MHC class I, and the 23% lower methylation of the BOLA class I histocompatibility antigen, alpha chain (Table 1C) in the SICTT positive cattle may preferentially lead to a CD8^+^ T cell response [40]. The gene showing the lowest levels of methylation is the Interleukin 1 receptor type 1 (*IL1R1*) with 46% lower methylation and of note, Interleukin-1 receptor-like 2 (*IL1RL2*) is also hypomethylated (19%) showing important regulation of the IL-1 signalling pathway by methylation in SICTT positive cattle. A smaller number of genes within the DMRs exhibit either exclusively CHH and CHG methylation alone or in combination with CG methylation. Three genes, namely Capping actin protein (*CAPZB*), the Retinoic acid receptor beta (*RARB*) and Kinesin family member 1 A (*KIF1A*) show all three types of methylation simultaneously. Interestingly, evidence has just emerged that the retinoic acid receptor pathway is exploited by mycobacteria for their survival [41] but the implications of multiple forms of cytosine methylation for gene expression remains unknown. A full list of all genes is given in Supplementary Table S4.

**Fig. 5:**
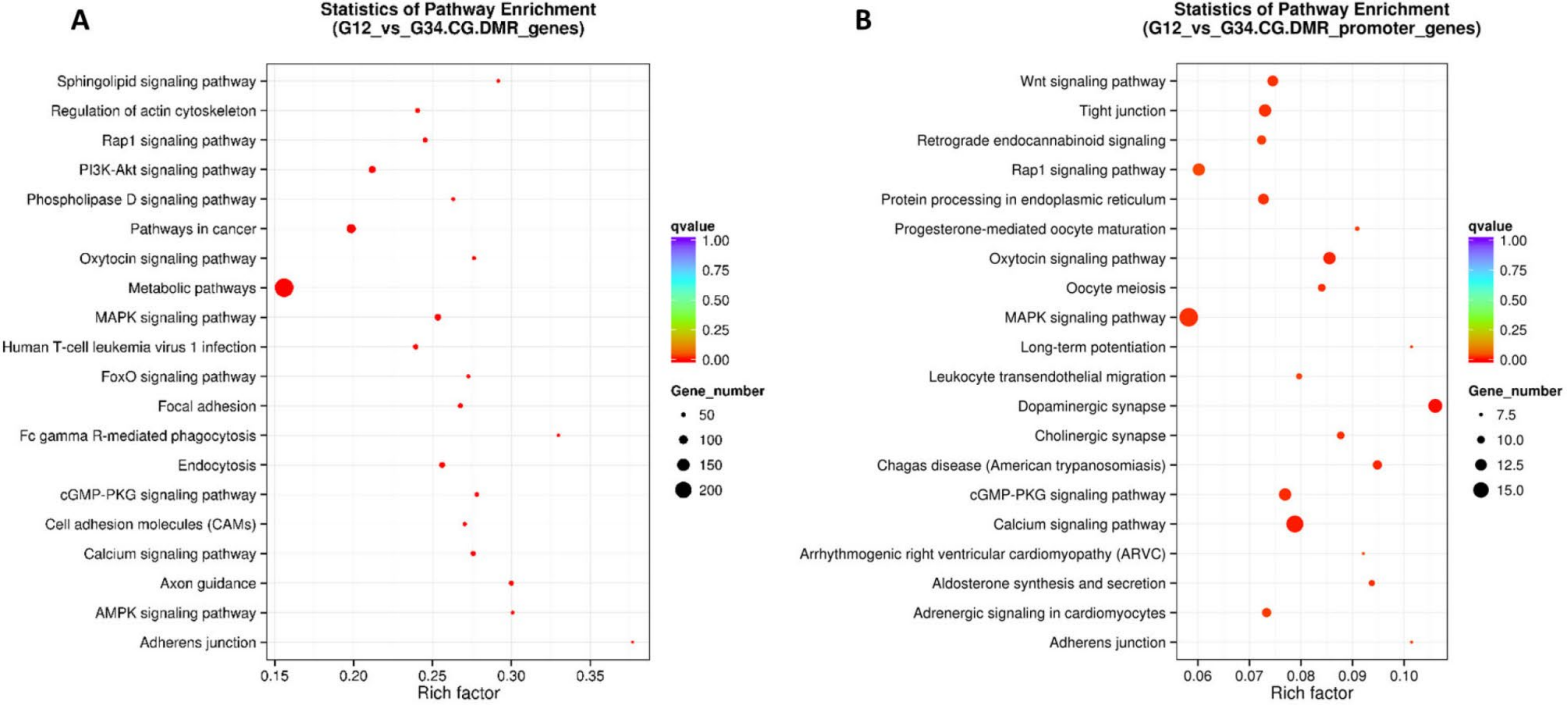
Scatterplot showing significantly enriched pathways from (A) DMRs and (B) DPMGs identified using KEGG. FDR corrected P values are indicated by the colour and the numbers of genes represented in the pathway is indicated by the size of the circle. The Rich factor is the ratio of differentially expressed gene numbers annotated in this pathway term relative to all gene numbers annotated in this pathway term

In order to identify pathways enriched by differentially methylated genes, GO and KEGG pathway analysis was conducted on genes identified in DMR and on DPMGs. No GO terms survived the P value adjustment (Supplementary Table S5), but 123 and 22 KEGG pathways were significantly enriched by genes in DMRs and DPMGs, respectively (FDR-*P* < 0.05). The top enriched pathways are shown in Table 2. The *MAPK signalling pathway* (bta04010) was the most significantly enriched by DMR genes (FDR-*P* = 4.29 × 10^7^), with *Calcium signalling pathway* (bta04020) significantly enriched by both DMR genes and DPMGs. Inhibition of calcium signalling has been identified as an immune evasive mechanism of *Mycobacterium tuberculosis* [42] and MAP kinases are also critical mediators of Interferon-γ production [43]. *Metabolic pathways* (bta01100) were also enriched by the greatest number of DMR genes (FDR-*P* = 2.4 × 10^5^) (Fig. 5). The enrichment of genes within the metabolic pathways including Inositol phosphate (bta00562), Sphingolipid (00600) and the mTOR signalling pathway (bta04150) also points towards important changes in the methylation of genes involved in nutrient partitioning which will ultimately impact on the efficacy of anti-mycobacterial immune responses [44]. A full list of the enriched KEGG pathways is shown in Supplementary Table S6.

**Table 1:** Differentially methylated genes in bTB + cows relative to test negative controls. (A) summary information on DMRs across exons and gene promoters (all data after line 2 uses a stringent *Diff Meth* minimum of 15%); (B) top annotated genes (ranked by Diff Meth) hypermethylated in bTB + cattle for DMR and DPMG datasets; (C) top annotated genes (ranked by Diff Meth) hypomethylated in bTB + cattle for DMR and DPMG datasets. Number of CpG sites (nCG); % Differential methylation (*% Diff Meth*) and areaStat (the sum of the test statistic of all CG sites within the DMR)

A							
**DMR summary details**				**Exons**		**Gene Promoters**	
Total number DMRs				1507		833	
Total number DMRs > 15%				223		159	
Hypermethylated DMRs (> 15%)				124		90	
Hypomethylated DMRs (>-15%)				99		69	
Minimum number consecutive CG per DMR				4		4	
Median number consecutive CG per DMR				18		12	
Maximum number consecutive CG per DMR				420		119	
Minimum DMR (%)				-0.49		-0.54	
Maximum DMR (%)				0.46		0.50	
Minimum DMR areaStat				-1779		-932	
Maximum DMR areaStat				4197		849	
**B**							
**Top ranked hypermethylated Genes in SICTT + cattle**							
*Ensembl ID*	*Gene Symbol*	*nCG*	*% Diff Meth*		*areaStat*		*Dataset*
ENSBTAG00000033107	*OSMR*	6	0.49		-55.75		DMR
ENSBTAG00000012921	*KIF24*	8	0.37		-60.47		DMR
ENSBTAG00000016679	*ETFDH*	6	0.35		-35.93		DMR
ENSBTAG00000018560	*DNAH3*	8	0.32		-54.42		DMR
ENSBTAG00000024751	*ULBP17*	36	0.3		-230.74		DMR
ENSBTAG00000014111	*INPP4B*	8	0.3		-55.17		DMR
ENSBTAG00000026080	*LAIR1*	16	0.26		-95.34		DMR
ENSBTAG00000054851	*OR2AG1G*	7	0.26		-33.65		DMR
ENSBTAG00000017021	*MCM4*	8	0.25		-58.57		DMR
ENSBTAG00000021195	*A1CF*	6	0.25		-32.62		DMR
ENSBTAG00000016722	*PGGHG*	10	0.54		-103.32		DPMG
ENSBTAG00000032538	*TPRG1*	4	0.5		-45.24		DPMG
ENSBTAG00000021077	*BOLA-DQB*	9	0.39		-47.82		DPMG
ENSBTAG00000009308	*TDRD3*	6	0.33		-38.13		DPMG
ENSBTAG00000000406	*GPATCH4*	6	0.32		-40.79		DPMG
ENSBTAG00000018743	*C5H12orf29*	6	0.3		-60.68		DPMG
ENSBTAG00000011767	*MPP5*	6	0.3		-34.91		DPMG
ENSBTAG00000017016	*H3F3C*	6	0.26		-30.26		DPMG
ENSBTAG00000017021	*MCM4*	8	0.25		-58.57		DPMG
ENSBTAG00000004147	*FBH1*	23	0.22		-105.17		DPMG
**C**							
**Top ranked hypomethylated Genes in SICTT + cattle**							
*Ensembl ID*	*Gene Symbol*	*nCG*	*% Diff Meth*		*areaStat*		*Dataset*
ENSBTAG00000005273	*IL1R1*	4	0.46		33.86		DMR
ENSBTAG00000022960	*HEPHL1*	8	0.29		45.85		DMR
ENSBTAG00000008814	*ADGRA2*	292	0.28		4196.67		DMR
ENSBTAG00000011447	*FAM171A2*	110	0.26		1102.27		DMR
ENSBTAG00000020566	*BCR*	16	0.24		107.74		DMR
ENSBTAG00000002069	*BOLA*	11	0.23		51.94		DMR
ENSBTAG00000053874	*NAT8L*	184	0.22		1737.83		DMR
ENSBTAG00000047491	*CACNA1S*	96	0.20		844.54		DMR
ENSBTAG00000046590	*FAM181A*	85	0.20		40.91		DMR
ENSBTAG00000032137	*PNPLA6*	7	0.19		757.12		DMR
ENSBTAG00000015190	*RMC1*	5	0.29		31.70		DPMG
ENSBTAG00000054342	*MIC1*	9	0.27		48.13		DPMG
ENSBTAG00000011940	*ZNF831*	16	0.25		145.92		DPMG
ENSBTAG00000022917	*FANK1*	20	0.23		144.85		DPMG
ENSBTAG00000017213	*MFAP1*	42	0.22		291.12		DPMG
ENSBTAG00000011249	*MOB2*	20	0.22		128.73		DPMG
ENSBTAG00000004168	*STON1*	12	0.19		58.31		DPMG
ENSBTAG00000020547	*BCAP31*	59	0.18		437.23		DPMG
ENSBTAG00000020551	*ABCD1*	59	0.18		437.23		DPMG
ENSBTAG00000033312	*ADAM3A*	19	0.18		118.05		DPMG

**Table 2:** The top-ranked biological pathways enriched for genes within differentially methylated regions (DMRs) and differentially promoter methylated genes (DPMGs) associated with *M. bovis* infection status assessed using KEGG

Pathway Name	ID	B-H *P*-value	Number of genes	Dataset
MAPK signalling pathway	Bta04010	4.59×10^− 7^	74	DMR
Axon guidance	Bta04360	4.59×10^− 7^	54	DMR
Calcium signalling pathway	Bta04020	1.88×10^− 6^	56	DMR
Endocytosis	Bta04144	2.0×10^− 6^	63	DMR
Focal adhesion	Bta04510	5.07×10^− 6^	53	DMR
Dopaminergic synapse	Bta04728	1.63×10^− 3^	14	DPMG
Calcium signalling pathway	Bta04020	5.64×10^− 3^	16	DPMG
Oxytocin signalling pathway	Bta04921	9.13×10^− 3^	13	DPMG
cGMP-PKG signalling pathway	Bta04022	1.45×10^− 2^	13	DPMG
Tight Junction	Bta04530	1.64×10^− 2^	13	DPMG

The number of differentially methylated genes identified in SICTT positive cattle in this study is similar to that recently reported for cattle with Johne’s disease [29]. Johne’s disease is caused by a related Mycobacterium sp. (*Myobacterium avium* subsp. *paratuberculosis*) and a total of 3,911, 4,336, and 4,094 DMGs were detected in clinical vs. subclinical, clinical vs. healthy, and subclinical vs. healthy groups, respectively. The same study reported some similarities in terms of differentially methylated genes to this current work (*IGF2* and *IGF1R*), as well as enriched *Calcium signalling pathways* which suggests that the two related diseases may share similar patho-epigenetic mechanisms [29].

## Comparison 2 - Differential methylation in SICTT negative cattle displaying antigen specific IFN-γ responses (IFN-γ positive)

**Fig. 6:**
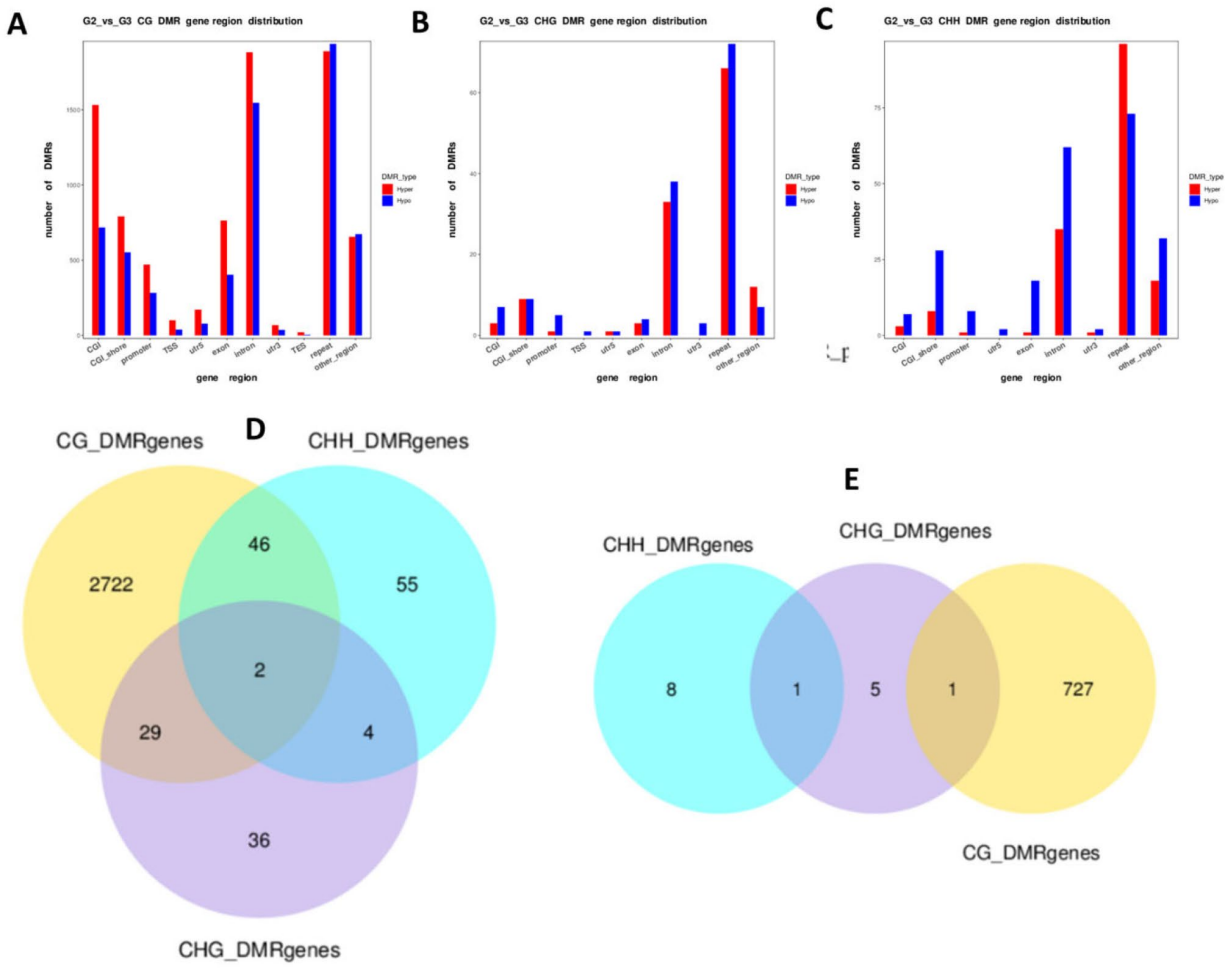
Numbers of genes identified as differentially methylated (CG, CHG or CHH) in (A) genes or (B) in the promoter region of genes of Group 2 (SICTT-/IFN-γ+) samples relative to Group 3 (SICTT+/IFG-γ+) samples. The degree of overlap shows the numbers of genes with multiple forms of methylation. The identity of these genes is given in Supplementary Table S8. The ratio of hypermethylation to hypomethylation in each of the CG, CHG and CHH sites is shown for each genomic feature in (B), (D) and (E) respectively

We then considered the methylation differences between Group 2 (SICTT-/IFN-γ+) samples relative to Group 3 (SICTT+/IFN-γ+) samples (*n* = 4/group). As IFN-γ positive cattle are considered at higher risk of bTB infection [12], we hypothesised that differential methylation at specific immune gene loci might influence the immune processes in these cattle. Considering the direction of methylation differences across genomic features, Group 2 samples showed an abundance of CG hypermethylation at most genomic features (Fig. 6A), a profile that was reduced or absent when examining CHG or CHH methylation patterns (Fig. 6B and Figure C, respectively). In addition, this profile contrasts with that shown when all SICTT positive and test negative controls are compared as shown in Fig. 3.

A total of 4,600 unique DMRs were identified between Group 2 (SICTT-/IFN-γ+) samples relative to Group 3 (SICTT+/IFN-γ+) samples across the genome (full list given in Supplementary Table S7). Within exons, 528 of which had a differential methylation value > 15%. These DMRs had a minimum length of 4 CG nucleotides and a maximum of 947 (median 29). The range in differential methylation across these sites was (-0.58 to 0.54) and the areaStat values varied from − 1384 to 9721. In gene promoters, differential methylation levels are higher (range − 0.61 to 0.65) with 823 DMRs in total, 358 with differential methylation value > 15%. Summary details for both exonic and promoter DMRs is given in Table 3A. In both exons and gene promoters, the numbers of hypermethylated sites exceeded that of hypomethylated sites (332 and 203, compared to 196 and 155 respectively, threshold > 15%) in Group 2 (SICTT-/IFN-γ+) samples relative to Group 3 (SICTT+/IFN-γ+) cattle (Table 3A). This hypermethylation is particularly evident in promoters and exonic genomic features in a CG context (Fig. 6A-C).

**Table 3:** Differentially methylated regions (DMRs) and differentially promoter methylated genes (DPMGs) in Group 2 (SICTT-/IFN-γ+) cows relative to Group 3 (SICTT+/IFN-γ+). (A) summary information on differentially methylated regions across exons and gene promoters (all data after line 2 uses a stringent *Diff Meth* minimum of 15%); (B) top 10 annotated genes (ranked by *Diff Meth*) located within DMRs that are differentially methylated in Group 2 cattle relative to Group 3; (C) top 10 annotated genes (ranked by *Diff Meth*) located within DPMGs that are differentially methylated in Group 2 cattle relative to Group 3. Number of CpG sites (nCG); % Differential methylation (*% Diff Meth*) and areaStat (the sum of the test statistic of all CG sites within the DMR)

A								
**DMR summary details**			**Exons**			**Gene Promoters**		
Total number DMRs			1211			823		
Total number DMRs > 15%			528			358		
Hypermethylated DMRs (> 15%)			332			203		
Hypomethylated DMRs (>-15%)			196			155		
Minimum number consecutive CG per DMR			4			4		
Median number consecutive CG per DMR			26			15		
Maximum number consecutive CG per DMR			947			947		
Minimum DMR (%)			-0.58			-0.61		
Maximum DMR (%)			0.54			0.65		
Minimum DMR areaStat			-1384			-964		
Maximum DMR areaStat			9722			9722		
**B**								
**Top ranked hypermethylated genes in Group 2 cattle**								
*Ensembl ID*	*Gene Symbol*	*nCG*		*% Diff Meth*	*areaStat*		*Dataset*	
ENSBTAG00000035915	*ABCA16*	4		0.54	29.92		DMR	
ENSBTAG00000013100	*SPAG5*	4		0.52	34.41		DMR	
ENSBTAG00000005273	*IL1R1*	4		0.43	24.75		DMR	
ENSBTAG00000008814	*ADGRA2*	286		0.41	4628.03		DMR	
ENSBTAG00000053730	*OR2F1*	8		0.41	63.81		DMR	
ENSBTAG00000046762	*MGC137098*	4		0.40	24.07		DMR	
ENSBTAG00000014910	*PIWIL4*	24		0.38	203.95		DMR	
ENSBTAG00000007567	*TMX3*	4		0.33	21.75		DMR	
ENSBTAG00000011447	*FAM171A2*	112		0.32	1179.92		DMR	
ENSBTAG00000015341	*DEPTOR*	44		0.32	374.46		DMR	
ENSBTAG00000008793	*RNASE1*	4		0.60	36.08		DPMG	
ENSBTAG00000049516	*ARL6IP1*	4		0.41	24.57		DPMG	
ENSBTAG00000052122	*OR5K22*	4		0.40	20.92		DPMG	
ENSBTAG00000051784	*OR5H33*	6		0.40	36.54		DPMG	
ENSBTAG00000024751	*ULBP17*	4		0.39	21.04		DPMG	
ENSBTAG00000011249	*MOB2*	8		0.39	51.29		DPMG	
ENSBTAG00000009725	*AOX1*	4		0.38	23.48		DPMG	
ENSBTAG00000049627	*OR52N4l*	10		0.37	55.56		DPMG	
ENSBTAG00000010304	*CHKA*	12		0.35	73.15		DPMG	
ENSBTAG00000015512	*HEXB*	6		0.34	36.05		DPMG	
**C**								
**Top ranked hypomethylated genes in Group 2 cattle**								
*Ensembl ID*	*Gene Symbol*	*nCG*		*% Diff Meth*	*areaStat*			*Dataset*
ENSBTAG00000017958	*AHI1*	4		0.58	-30.86			DMR
ENSBTAG00000012921	*KIF24*	6		0.50	-49.36			DMR
ENSBTAG00000049903	*OR4G10*	4		0.44	-22.73			DMR
ENSBTAG00000008032	*ACTR3B*	4		0.43	-21.76			DMR
ENSBTAG00000021876	*WDR72*	4		0.34	-22.98			DMR
ENSBTAG00000038858	*CKLF*	103		0.32	-964.16			DMR
ENSBTAG00000014494	*RNASET2*	46		0.31	-338.01			DMR
ENSBTAG00000007547	*CACNG1*	116		0.31	-1254.21			DMR
ENSBTAG00000000697	*RRP8*	18		0.31	-115.92			DMR
ENSBTAG00000016835	*IL17F*	22		0.30	-130.59			DMR
ENSBTAG00000017958	*AHI1*	4		0.58	-30.86			DPMG
ENSBTAG00000016722	*PGGHG*	10		0.54	-67.60			DPMG
ENSBTAG00000042584	*RF00026*	5		0.53	-39.77			DPMG
ENSBTAG00000020301	*BLM*	4		0.51	-26.65			DPMG
ENSBTAG00000049903	*OR4G10*	4		0.44	-22.73			DPMG
ENSBTAG00000009308	*TDRD3*	5		0.44	-29.98			DPMG
ENSBTAG00000029988	MIR331	4		0.43	-21.66			DPMG
ENSBTAG00000032538	*TPRG1*	4		0.42	-24.35			DPMG
ENSBTAG00000018743	*C5H12orf29*	7		0.41	-68.88			DPMG
ENSBTAG00000029683	*U1*	4		0.41	-22.46			DPMG

The number of genes identified within the DMRs and DPMGs is shown in Fig. 6D and E. As previously, the majority of these genes (2722 exonic genes and 727 promoter genes) are CG methylated the top ranked differentially methylated genes in DMRs and DPMGs shown in Table 3B and Table 3C. Amongst these is the Interleukin 1 receptor (*IL1R1*) with 43% higher methylation in Group 2 cattle which may affect inflammatory signalling in these cattle. In addition, BOLA class I histocompatibility antigen, alpha chain (*BOLA*) is also hypermethylated (25%) in Group 2 samples, which may alter CD8^+^ T cell responses. Interestingly, the gene encoding the DEP domain containing MTOR interacting protein (*DEPTOR*) exhibits 32% higher methylation in Group 2 cattle, which may negatively regulate kinase activity [45]. The Interleukin-10 receptor subunit alpha gene (*IL10RA*) is also hypermethylated by 22% indicating a potential reduction in the regulatory effects of this cytokine. In contrast, the gene encoding Interleukin 17F (*IL17F*) is hypomethylated in Group 2 samples by 30% (Table 3C). IL-17 is a crucial cytokine with potent inflammatory and anti-mycobacterial functions [46] and hypomethylation suggests increased expression in Group 2 cattle. Alongside IL-17A, IL-17F has been shown to be potently induced in response to mycobacterial antigens in *M. bovis* infected cattle [47] and is associated with a protective response, including post-vaccination. Host defence genes with well characterised antimicrobial and immunomodulatory roles during mycobacterial infection [48] including defensin genes (*BDEF109*) and (*DEFB*), which were hypomethylated by 31% and 18% respectively.

Short, stable, microRNAs (miRNA) have also been investigated as potential biomarkers for multiple mycobacterial infections in cattle and they are proposed to have stage specificity for disease diagnosis [49]. DMRs identified in this study encompass multiple regions regulating miRNA expression which may offer insights into potential diagnostics to identify similar Group 2 cattle. The following miRNA are hypermethylated in Group 2 cattle (*bta-mir-126* (17%), *bta-mir-2447* (22%), *bta-mir-2285ab* (34%), *bta-mir-11,993* (15%), *bta-mir-2385* (16%), *bta-mir-2309* (20%), *bta-mir-7861* (24%). In contrast, others are hypomethylated (*bta-mir-671* (16%), *bta-mir-2887-2* (39%), and *bta-mir-331* is hypomethylated by 43% (Table 3C and Supplementary Table S7). A smaller number of genes within the DMRs exhibit either exclusively CHH and CHG methylation alone or in combination with CG methylation (Supplementary Table S8).

To investigate the potential associations of the DMGs and DPMGs with IFN-γ status, functional enrichment analysis was performed. While only a single GO term survived the P value adjustment for multiple testing, identifying enrichment of the molecular function ‘*Binding*’ (GO:0005488, FDR-*P* = 9.1 × 10^3^) (Supplementary Table S9). In contrast, 132 and 2 KEGG pathways were enriched by identified DMR genes and DPMGs including the *Calcium signalling pathway* (FDR-*P* = 2.86 × 10^− 9^) and the *MAPK signalling pathway* (FDR-*P* = 2.11 × 10^− 7^) (Table 4) which are of major relevance to mycobacterial infection. The *Metabolic signalling pathway* contained an input of 205 genes and was significantly enriched (FDR-*P* = 5.23 × 10^− 8^). Other significantly enriched pathways include the *NFkB signalling pathway* (bta04064, FDR-*P* = 3.7 × 10^− 3^) and *Th17 cell differentiation pathway* (bta04659, FDR-*P* = 2.9 × 10^− 3^). A full list of the enriched KEGG pathways is shown in Supplementary Table S10. For reference, data for all pairwise comparisons of the experimental groups shown in Fig. 1 is supplied in Supplementary Figures S2–S6.

**Table 4:** The top-ranked biological pathways enriched for genes within differentially methylated regions (DMRs) and differentially promoter methylated genes (DPMGs) associated with Group 2 (SICTT-/IFN-γ+) cows relative to Group 3 (SICTT+/IFN-γ+) cows assessed using KEGG.

Pathway name	ID	B-H *P*-value	Number of genes	dataset
Calcium signalling pathway	Bta04020	2.86×10^− 9^	58	DMR
MAPK signalling pathway	Bta04010	2.11×10^− 7^	66	DMR
Oxytocin signalling pathway	Bta04921	2.11×10^− 7^	44	DMR
Metabolic pathway	Bta01100	2.93×10^− 6^	205	DMR
Axon Guidance	Bta04360	2.93×10^− 6^	45	DMR
Long term potentiation	Bta04720	4.00 X 10^− 2^	8	DPMG
Calcium signalling pathway	Bta04020	4.00 X 10^− 2^	14	DPMG

## Conclusions

Deciphering methylation patterns across the entire genome is complex, and consequently the understanding of the epigenome in livestock species is in its early stages. Current diagnostics for bTB rely largely on tuberculin skin test responses as a measure of the probability of infection and it is plausible that atypical immune responses in some cattle may contribute to the imperfect sensitivity and specificity of these tests. Under natural infection conditions where physiological demands on cattle are acute and concurrent infections often occur, aberrant methylation of DNA might affect immune responses, diagnostics and disease outcomes. In addition, mycobacteria share a long evolutionary history with their respective hosts, so it is not unexpected that differential methylation may be manipulated by pathogens to enhance their survival [22, 23]. In this study we have identified extensive differential methylation of host DNA, specifically of genes regulating *MAPK*, *Calcium* and *Metabolism signalling pathways* in cattle with different SICTT test results and identified specific immune gene loci that could potentially affect immune responses and diagnostic test performance. This study provides a new layer of understanding to the complexities of host-pathogen interaction during infection with *Mycobacterium bovis* [50]. However, consensus on the optimal thresholds for filtering whole genome methylation data has yet to be agreed [51] and as the sample size in the current study is limited, further detailed validation of disease-associated methylation changes in target genes in larger populations will be required to identify specific loci associated with infection which may affect diagnostic test performance.

## Materials and methods

### Cattle tuberculin skin test, IFN-γ assay and DNA extraction

The SICTT was carried out by intradermal injection of cattle with 0.1 mL PPD-bovine and PPD-avian (Thermo-Fisher Scientific) at sites 12 cm apart in the mid-neck region using a McLintock tuberculin syringe. Skin thicknesses were measured in mm at both sites before the intradermal injection and after 72 h in accordance with Council Directive 64/432/EEC (2015) and OIE (2009). Based on the results of the SICTT, the animal was defined as a standard reactor if the bovine reaction was both positive and exceeded the avian reaction by > 4 mm; as a standard inconclusive reactor if the bovine reaction was either positive or inconclusive, > 1–4 mm above the avian reaction. Peripheral blood was collected via the coccygeal vein in heparin-treated vacutainers from known bTB infected herds maintained by the Irish Department of Agriculture, Food and the Marine (DAFM) as part of the national bTB control programme and were not euthanised as part of this study. Farmer consent was therefore not required. Blood samples were stimulated with PPDb and PPDa tuberculin (Thermo-Fisher Scientific) within 8 h of blood collection. Gamma-Interferon (IFN-γ) production was measured in duplicate samples by ELISA using a semi-quantitative commercial diagnostic kit according to kit instructions (Bovigam, Thermo-Fisher Scientific). Absorbance values at OD_450_ nm were converted to OD units using the formula, OD450 × 1000. A sample was positive when the OD_450_ of the PPDb stimulated sample exceeded 100 OD units (PPDb > 100), was greater than the nil un-stimulated sample by 50 OD units (PPDb - Nil sample > 50) and was greater than the PPDa stimulated sample (PPDb - PPDa > 80). These interpretation criteria are used in the Irish national bTB eradication programme [10].

Sixteen animals were chosen as either positive (*n* = 8) or negative (*n* = 8) on the single intradermal comparative tuberculin test (SICTT). Among these, four animals in each group were also positive on the IFN-γ ELISA (Supplementary Table S1). In follow-up of animals after the study was completed, all but two of the animals had been euthanised. Post-mortem examination at the abattoir revealed that three animals (2 x SICTT positive / IFN-γ positive, 1 x SICTT positive / IFN-γ negative) disclosed caseous lesions in the retropharyngeal lymph nodes, consistent with *M. bovis* infection. Ethical approval for their use in this study was provided by the UCD ethics committee under licence number AREC-E-22-33-Meade. Genomic DNA was subsequently extracted from frozen whole blood representing 16 Friesian cows as shown in Fig. 1. A full list of test results for each animal is shown in Supplementary Table S1. DNA was extracted using the QIAamp Blood Mini Kit (Qiagen) and the purity of the extracted DNA was assessed using the 260/280 ratio.

### Bisulfite treatment and WGBS library preparation

The EZ DNA Methylation Gold Kit (Cat No. D5005) Zymo Research was used to perform the bisulfite treatment of the unmethylated cytosines present in extracted DNA samples. Cluster generation and high-throughput sequencing of post-bisulfite adaptor tagging (PBAT) libraries were performed using an Illumina^®^ NovaSeq™ 6000 sequencing system on an S4 flow cell with paired-end 150 bp reads using the Illumina S4 reagent kit v1.5, by Novogene (UK) Company Ltd., Cambridge. Uniquely indexed samples were pooled for partial lane sequencing with demultiplexing and initially run to generate one Gigabase (Gb) of sequencing data (approximately 3.3 M PE150 reads) to assess the cytosine conversion ratio. Subsequently, samples with unique indices were pooled for further partial lane sequencing with demultiplexing until approximately 90 Gb (300 M PE150 reads) of data per sample was generated.

### Data quality control, estimation of methylation levels and identification of differential methylated regions (DMRs)

FastQC (fastqc_v0.11.5) was used to perform basic statistics assessing the quality of the raw reads. Read sequences produced by the Illumina pipeline in FASTQ format were subsequently pre-processed through Trimmomatic (Trimmomatic-0.36) software using the parameter (SLIDINGWINDOW: 4:15; LEADING:3, TRAILING:3; ILLUMINACLIP: adapter.fa: 2:30:10; MINLEN:36). Reads that passed all the filtering steps (clean reads) were used for all subsequent analyses. FastQC was used to perform basic quality statistics on the clean data reads. Prior to the analysis, reference data for the bovine, were assembled which included the reference sequence fasta file, the annotation file in gtf format, the GO annotation file, the description file and the gene region file in bed format. For the bed files, repeat regions were predicted using RepeatMasker, followed by using a CGI track from a genome using cpgIslandExt. Bismark software (version 0.16.3; [52]) was used to perform alignments of bisulfite-treated reads to the bovine reference genome (-X 700 --dovetail). The reference genome was firstly transformed into a bisulfite-converted version (C-to-T and G-to-A converted) and then indexed using bowtie2 software [53]. Sequence reads were also transformed into bisulfite-converted versions (C-to-T and G-to-A converted) before they were aligned to similarly converted versions of the bovine genome in a directional manner. Sequence reads that produced a unique best alignment from the two alignment processes (top and bottom strand) were then compared to the normal genomic sequence and the methylation state of all cytosine positions in the read was inferred. The same reads that aligned to the same regions of genome were regarded as duplicated. The sequencing depth and coverage were summarized using de-duplicated reads. Results from methylation extractor (bismark_methylation_extractor, -- no_overlap) were transformed into bigWig format for visualization using the IGV browser. The sodium bisulfite non-conversion rate was calculated as the percentage of cytosine sequenced at cytosine reference positions in the lambda genome. To identify the methylation site, the sum Mc of methylated counts was modelled as a binomial (Bin) random variable with methylation rate. In order to calculate the methylation level, the sequence was divided into multiple bins (bin size of 10 kb) and the sum of methylated and unmethylated read counts in each window calculated. Methylation level (ML) for each window or C site represents the fraction of methylated Cs and was defined as: ML(C) = reads (mC)/reads (mC) + reads (C). Differentially methylated regions (DMRs) were identified using DSS software [54–56] and the core of DSS is a new dispersion shrinkage method for estimating the dispersion parameter from Gamma-Poisson or Beta-Binomial distributions. DSS possesses three characteristics to detect DMRs: firstly, spatial correlation and proper utilization of the information from neighbouring cytosine sites improves the estimation of ML at each C site, and hence improve DMR detection; secondly, the read depth of the C sites provides information on precision used to improve statistical tests for DMR detection; and thirdly, the variance among biological replicates provides information necessary for a valid statistical test to detect DMRs. According to the distribution of DMRs through the genome, genes related to DMRs were defined as genes whose gene body region (from TSS to TES) or promoter region (upstream 2 kb from the TSS) have an overlap with the DMRs.

### Gene ontology and pathway analysis

Gene Ontology (GO) enrichment analysis of genes related to DMRs was implemented by the GOseq R package [55], in which gene length bias was corrected and GO terms with corrected FDR P-value < 0.05 were considered significantly enriched by DMR-related genes. KOBAS software [57] was used to test the statistical enrichment of DMR-related genes using KEGG [58]. Bioinformatic analysis was performed by the Novogene bioinformatics team and the described methods herein represent a common data analysis pipeline described similarly in other studies and which were provided by Novogene (www.novogene.com).

### Electronic supplementary material

Below is the link to the electronic supplementary material.

**Supplementary Material 1**

**Supplementary Material 2 - Supplementary Table S1:** Single intradermal comparative tuberculin test (SICTT), IFN-γ release assay results, bTB classification and sampling information on all cattle sampled

**Supplementary Material 3 - Supplementary Table S2:** Bisulphite conversion data, genome mapping statistics, % methylation by sample and by genomic feature for 16 WGBS libraries from *M. bovis*-infected and non-infected control cattle

**Supplementary Material 4 (A and B)- Supplementary Figure S1 (A and B):** Methylation profiles for all 16 WGBS samples across genomic features (A) and genes (B). For group information on sample ID, refer to Table S1

**Supplementary Material 5 - Supplementary Table S3:** Differentially expressed regions (DMRs) across all genomic features from *M. bovis*-infected and non-infected control animals is shown in Tab 1. Subsequent tabs are subsets of this data including DMRs in exons and promoters with >15% differential methylation

**Supplementary Material 6 - Supplementary Table S4:** Differentially expressed promoter region genes (DMPGs) in various genomic features from *M. bovis*-infected and non-infected control animals. Genes identified with CH, CHH or CHG methylation types are listed with Ensembl ID and Gene Symbols. The overlap tab lists genes with multiple methylation types

**Supplementary Material 7 - Supplementary Table S5:** GO enrichment analysis showing enriched GO terms (biological processes, cellular component and molecular function) for both DMR and DPMG genes from *M. bovis*-infected and non-infected control animals

**Supplementary Material 8 - Supplementary Table S6:** KEGG analysis showing enriched canonical pathways for both DMR and DPMG genes from *M. bovis*-infected and non-infected control animals

**Supplementary Material 9 - Supplementary Table S7:** Differentially expressed regions (DMRs) across all genomic features from Group 2 (SICTT-/IFNG+) samples relative to Group 3 (SICTT+/IFNG+) samples is shown in Tab 1. Subsequent tabs are subsets of this data including DMRs in exons and promoters with >15% differential methylation

**Supplementary Material 10 - Supplementary Table S8:** Differentially expressed promoter region genes (DMPGs) in various genomic features from Group 2 (SICTT-/IFNG+) samples relative to Group 3 (SICTT+/IFNG+) samples. Genes identified with CH, CHH or CHG methylation types are listed with Ensembl ID and Gene Symbols. The overlap tab lists genes with multiple methylation types

**Supplementary Material 11 - Supplementary Table S9:** GO enrichment analysis showing enriched GO terms (biological processes, cellular component and molecular function) for DMRs and DPMGs for Group 2 (SICTT-/IFNG+) samples relative to Group 3 (SICTT+/IFNG+) samples (as shown in Figure 1)

**Supplementary Material 12 - Supplementary Table S10:** KEGG analysis showing enriched canonical pathways for DMRs and DPMGs for Group 2 (SICTT-/IFNG+) samples relative to Group 3 (SICTT+/IFNG+) samples (as shown in Figure 1)

**Supplementary Material 13 - Figure S2:** Differential methylation profiles for all 16 WGBS samples, divided according to experimental groups (as shown in Figure 1). Methylation plots are shown for combined levels of CG, CHG and CHH methylation types as well as each type individually for pairwise each group comparison

**Supplementary Material 14 - Figure S3:** Ratio of hypermethylation to hypomethylation profiles for all 16 WGBS samples, divided according to experimental groups (as shown in Figure 1) across each genomic feature. Histograms show ratio for CG, CHG and CHH methylation types for each pairwise group comparison

**Supplementary Material 15 - Figure S4:** Numbers of genes identified as differentially methylated (CG, CHG or CHH) in DMR regions and in the promoter region of genes for all 16 WGBS samples, divided according to experimental groups (as shown in Figure 1). The degree of overlap shows the numbers of genes with multiple forms of methylation

**Supplementary Material 16 - Figure S5:** GO plot enrichment of biological processes, cellular component and molecular function for all 16 WGBS samples, divided according to experimental groups (as shown in Figure 1). Significantly enriched GO categories are identified with an asterix

**Supplementary Material 17 - Figure S6:** Scatterplot showing significantly enriched pathways from DMRs and DPMGs for all 16 WGBS samples, divided according to experimental groups (as shown in Figure 1) identified using KEGG. Corrected P values (Q value) is indicated by the colour and the numbers of genes represented in the pathway is indicated by the size of the circle. The Rich factor identifies the ratio of differentially expressed gene numbers annotated in this pathway term relative to all gene numbers annotated in this pathway term

## Data Availability

The data sets presented in this study can be found in online repositories. The names of the repository/repositories and accession number(s) can be found at: www.ebi.ac.uk/biostudies/arrayexpress/studies/E-MTAB-13487.
