# Supplementary material for: Extensive differential DNA methylation between tuberculosis skin test positive and skin test negative cattle
Source: BMC Genomics. 2024 Aug 6;25:762. doi: 10.1186/s12864-024-10574-x (PMC11301934; doi:10.1186/s12864-024-10574-x)
Supplement: Supplementary file 1 — Supplementary Material 1 [file 12864_2024_10574_MOESM1_ESM.docx]

**Supplementary Material File 1**

**Extensive Differential DNA Methylation in Bovine Tuberculosis Skin Test Positive and Skin Test Negative Cattle**

Sajad A. Bhat^1^, Alia Parveen^1^, Eamonn Gormley^2^ and Kieran G. Meade^1, 3, 4^.

^1^ UCD School of Agriculture and Food Science, University College Dublin, Belfield, D04 V1W8, Dublin, Ireland

^2^ UCD School of Veterinary Medicine, University College Dublin, Belfield, D04 V1W8, Dublin, Ireland

^3^ UCD Conway Institute of Biomolecular and Biomedical Research, University College Dublin, Belfield, D04 V1W8, Dublin, Ireland

^4^ UCD Institute of Food and Health, University College Dublin, Belfield, C15 PW93, Dublin, Ireland.

Correspondence

K. G. Meade, UCD School of Agriculture and Food Science, University College Dublin, Belfield, Dublin, D04 V1W8, Ireland.

Email: kieran.meade@ucd.ie

Phone: +353 1 716 6925

**Supplementary Material File 2 - Supplementary Table S1:** Single intradermal comparative tuberculin test (SICTT), IFN-γ release assay results, bTB classification and sampling information on all cattle sampled.

**Supplementary Material File 3 - Supplementary Table S2**: Bisulphite conversion data, genome mapping statistics, % methylation by sample and by genomic feature for 16 WGBS libraries from *M. bovis*-infected and non-infected control cattle.

**Supplementary Material File 4- Supplementary Figure S1 (A and B):** Methylation profiles for all 16 WGBS samples across genomic features (A) and genes (B). For group information on sample ID, refer to Table S1.

**Supplementary Material File 5 - Supplementary Table S3**: Differentially expressed regions (DMRs) across all genomic features from *M. bovis*-infected and non-infected control animals is shown in Tab 1. Subsequent tabs are subsets of this data including DMRs in exons and promoters with >15% differential methylation.

**Supplementary Material File 6 - Supplementary Table S4**: Differentially expressed promoter region genes (DMPGs) in various genomic features from *M. bovis*-infected and non-infected control animals. Genes identified with CH, CHH or CHG methylation types are listed with Ensembl ID and Gene Symbols. The overlap tab lists genes with multiple methylation types.

**Supplementary Material File 7 - Supplementary Table S5**:: GO enrichment analysis showing enriched GO terms (biological processes, cellular component and molecular function) for both DMR and DPMG genes from *M. bovis*-infected and non-infected control animals.

**Supplementary Material File 8 - Supplementary Table S6**: KEGG analysis showing enriched canonical pathways for both DMR and DPMG genes from *M. bovis*-infected and non-infected control animals.

**Supplementary Material File 9 - Supplementary Table S7**: Differentially expressed regions (DMRs) across all genomic features from Group 2 (SICTT-/IFNG+) samples relative to Group 3 (SICTT+/IFNG+) samples is shown in Tab 1. Subsequent tabs are subsets of this data including DMRs in exons and promoters with >15% differential methylation.

**Supplementary Material File 10 - Supplementary Table S8**: Differentially expressed promoter region genes (DMPGs) in various genomic features from Group 2 (SICTT-/IFNG+) samples relative to Group 3 (SICTT+/IFNG+) samples. Genes identified with CH, CHH or CHG methylation types are listed with Ensembl ID and Gene Symbols. The overlap tab lists genes with multiple methylation types.

**Supplementary Material File 11 - Supplementary Table S9**: GO enrichment analysis showing enriched GO terms (biological processes, cellular component and molecular function) for DMRs and DPMGs for Group 2 (SICTT-/IFNG+) samples relative to Group 3 (SICTT+/IFNG+) samples (as shown in Figure 1).

**Supplementary Material File 12 - Supplementary Table S10**: KEGG analysis showing enriched canonical pathways for DMRs and DPMGs for Group 2 (SICTT-/IFNG+) samples relative to Group 3 (SICTT+/IFNG+) samples (as shown in Figure 1).

**Supplementary Material File 13 - Supplementary Figure S2:** Differential methylation profiles for all 16 WGBS samples, divided according to experimental groups (as shown in Figure 1). Methylation plots are shown for combined levels of CG, CHG and CHH methylation types as well as each type individually for pairwise each group comparison.

**Supplementary Material File 14 - Supplementary Figure S3:** Ratio of hypermethylation to hypomethylation profiles for all 16 WGBS samples, divided according to experimental groups (as shown in Figure 1) across each genomic feature. Histograms show ratio for CG, CHG and CHH methylation types for each pairwise group comparison.

**Supplementary Material File 15 - Supplementary Figure S4:** Numbers of genes identified as differentially methylated (CG, CHG or CHH) in DMR regions and in the promoter region of genes for all 16 WGBS samples, divided according to experimental groups (as shown in Figure 1). The degree of overlap shows the numbers of genes with multiple forms of methylation.

**Supplementary Material File 16 - Supplementary Figure S5:** GO plot enrichment of biological processes, cellular component and molecular function for all 16 WGBS samples, divided according to experimental groups (as shown in Figure 1). Significantly enriched GO categories are identified with an asterix.

**Supplementary Material File 17 - Supplementary Figure S6:** Scatterplot showing significantly enriched pathways from DMRs and DPMGs for all 16 WGBS samples, divided according to experimental groups (as shown in Figure 1) identified using KEGG. Corrected P values (Q value) is indicated by the colour and the numbers of genes represented in the pathway is indicated by the size of the circle. The Rich factor identifies the ratio of differentially expressed gene numbers annotated in this pathway term relative to all gene numbers annotated in this pathway term.
