# Supplementary figures and images for: Extensive differential DNA methylation between tuberculosis skin test positive and skin test negative cattle
Source: BMC Genomics. 2024 Aug 6;25:762. doi: 10.1186/s12864-024-10574-x (PMC11301934; doi:10.1186/s12864-024-10574-x)

| 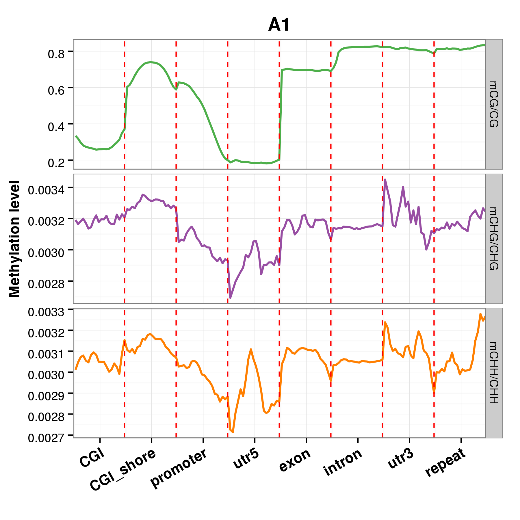 | 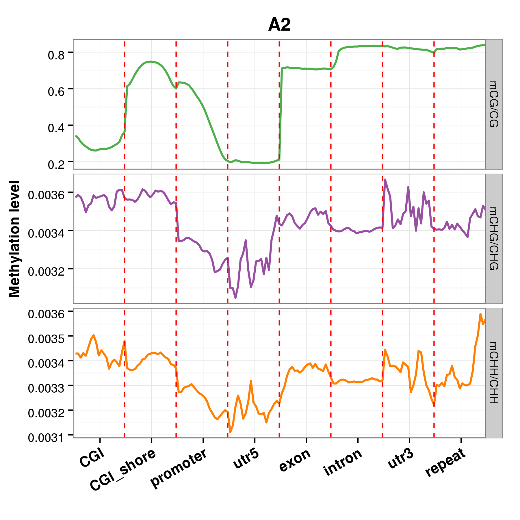 | 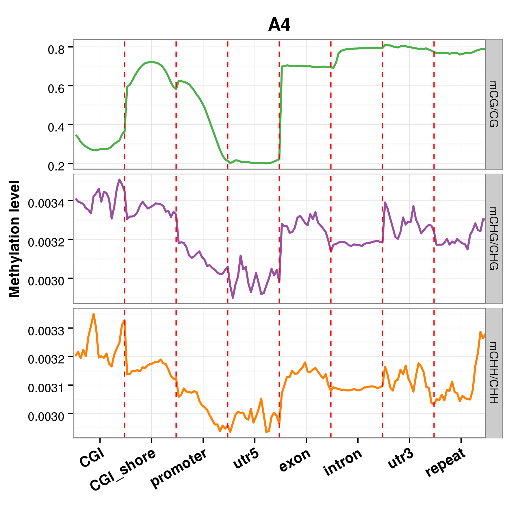 | 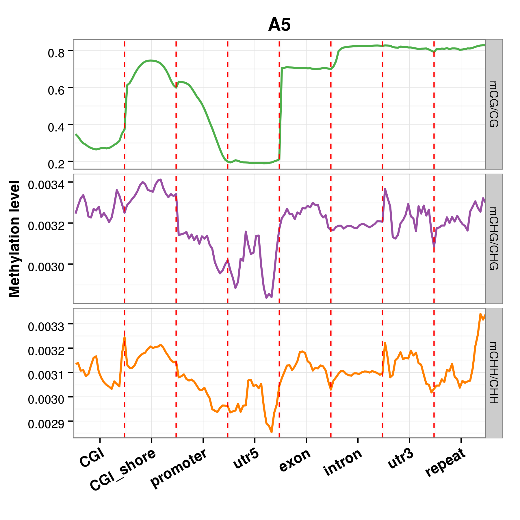 |
| --- | --- | --- | --- |
| 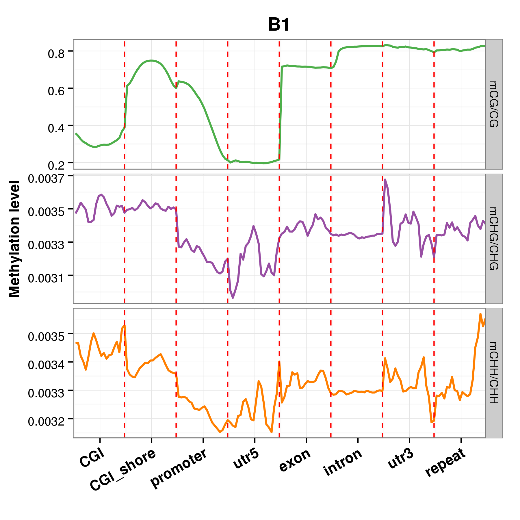 | 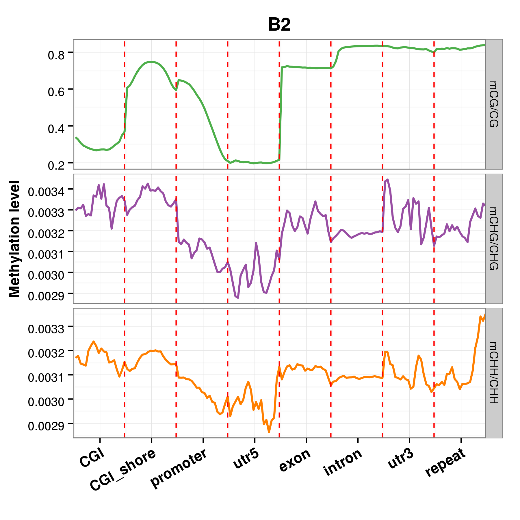 | 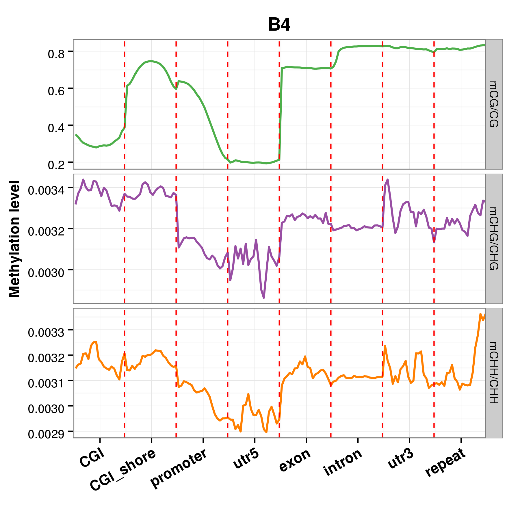 | 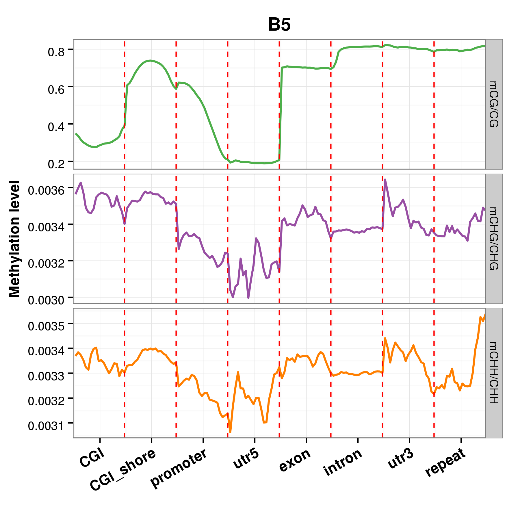 |
| 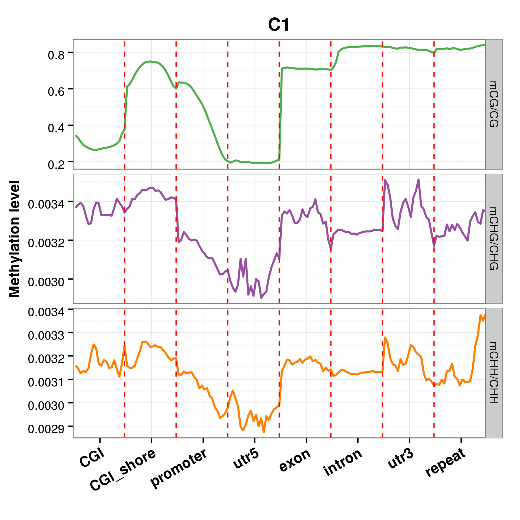 | 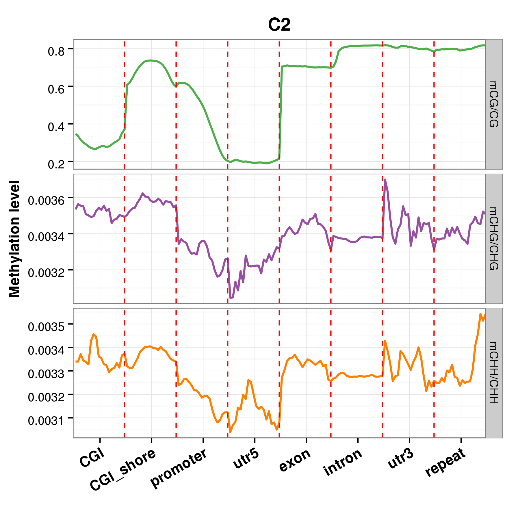 | 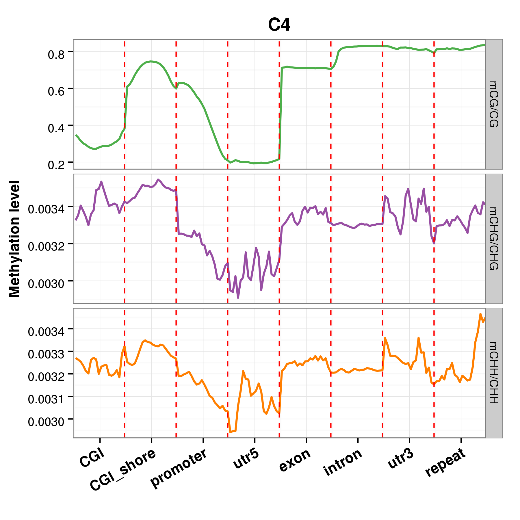 | 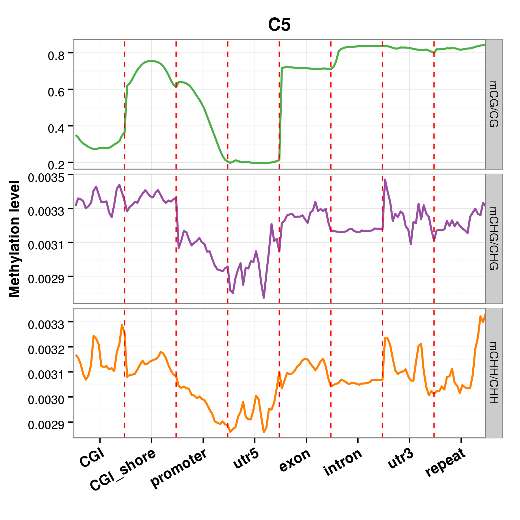 |
| 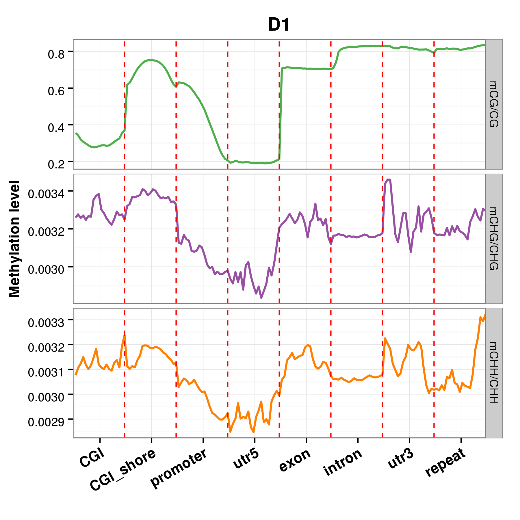 | 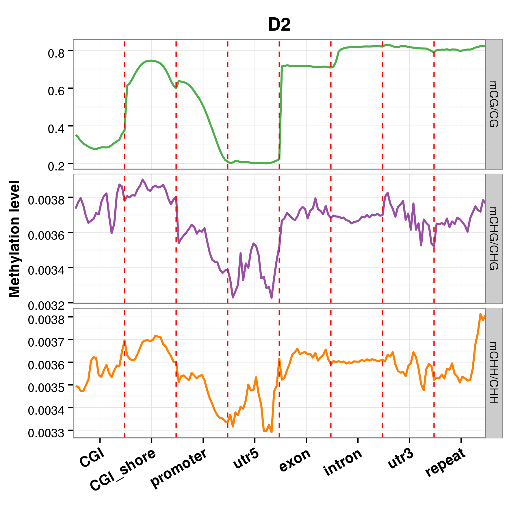 | 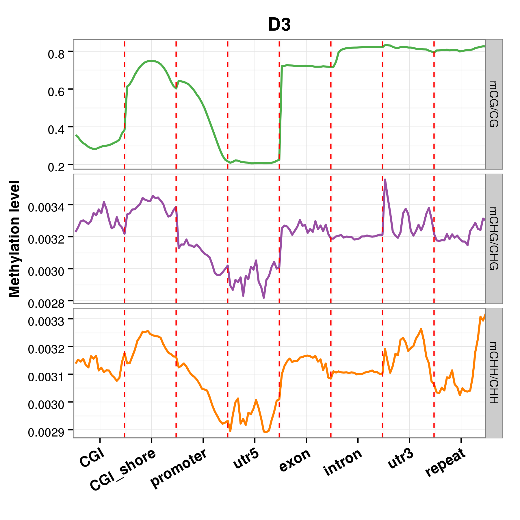 | 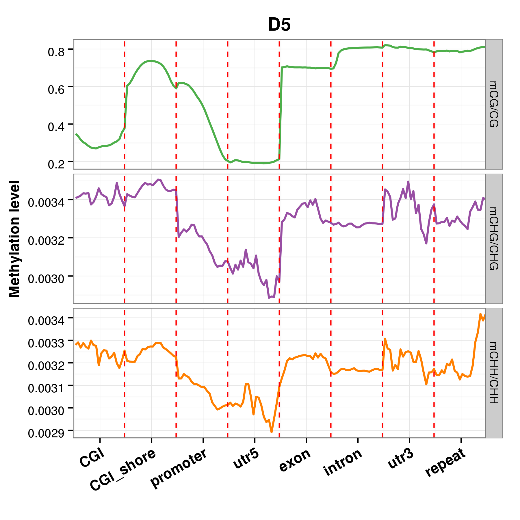 |

| 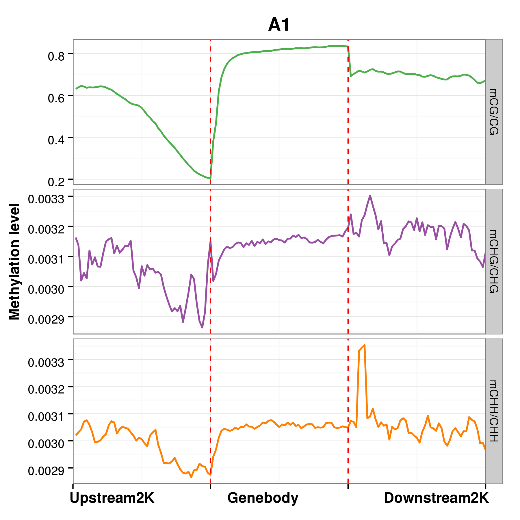 | 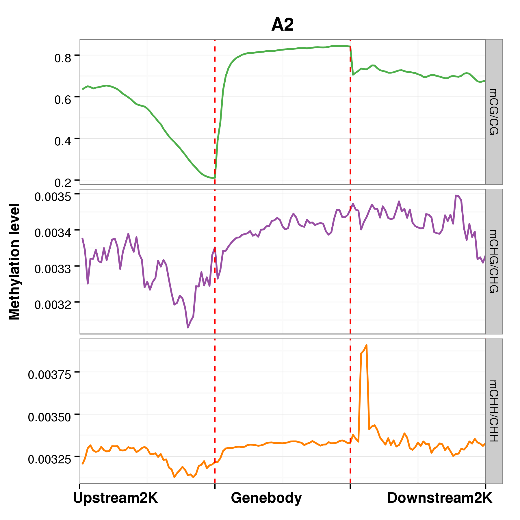 | 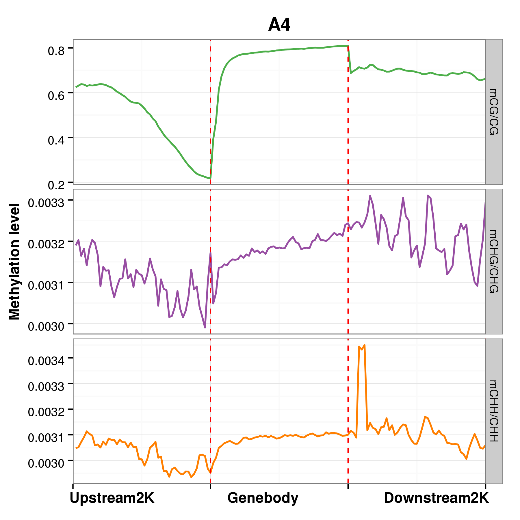 | 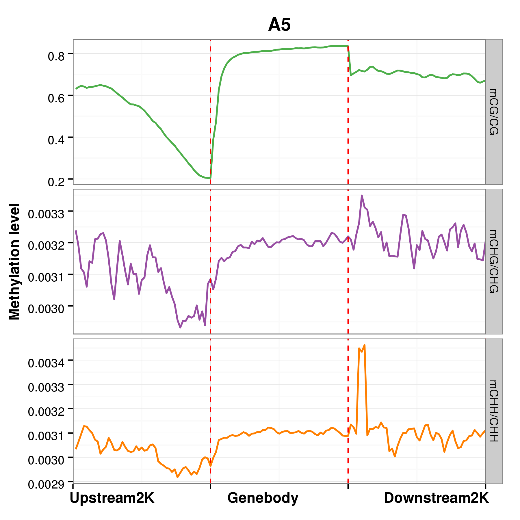 |
| --- | --- | --- | --- |
| 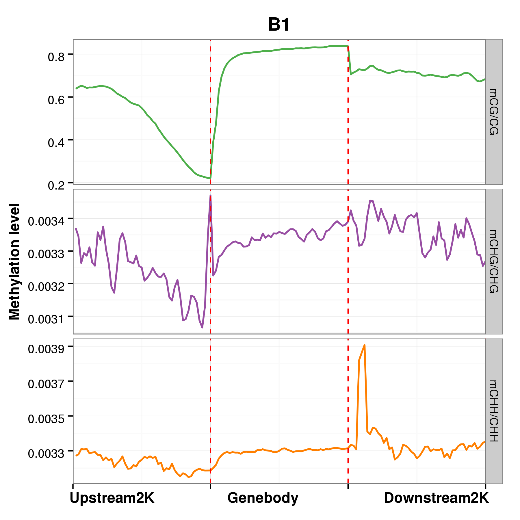 | 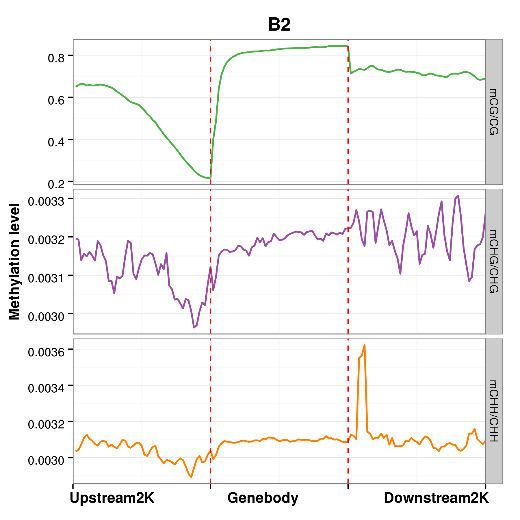 | 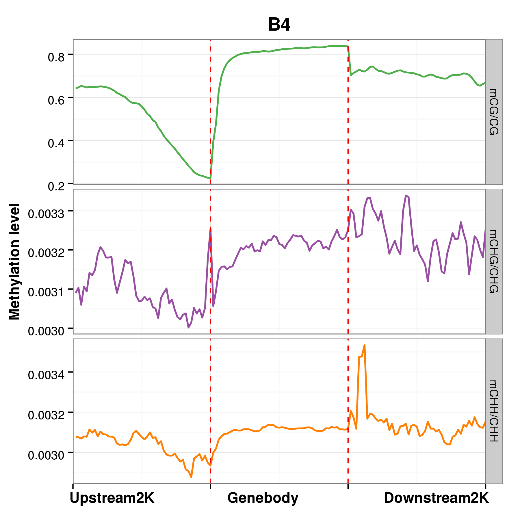 | 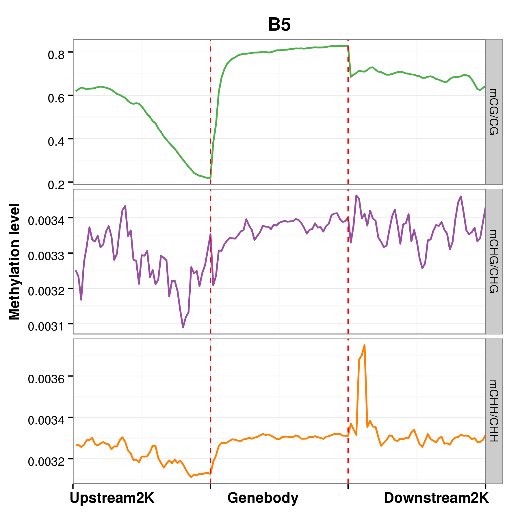 |
| 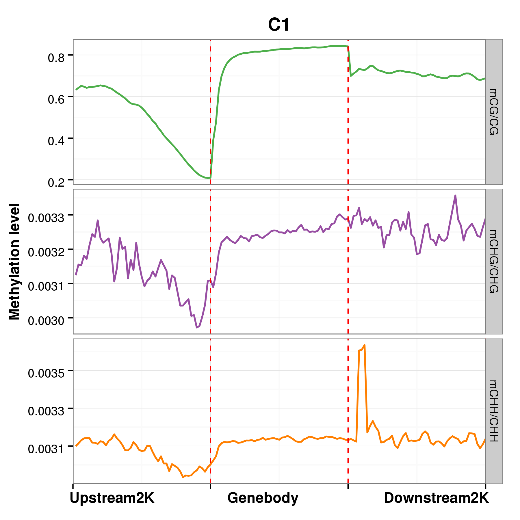 | 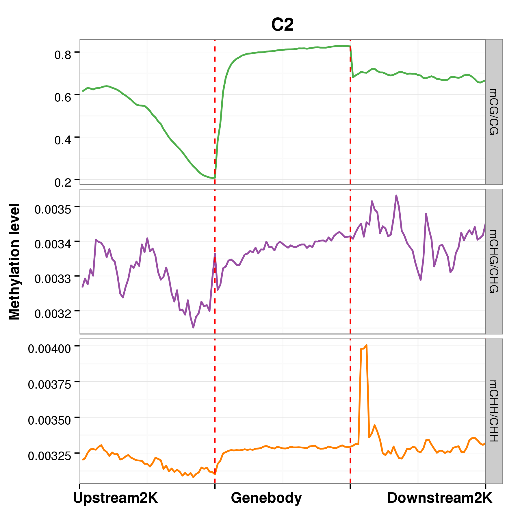 | 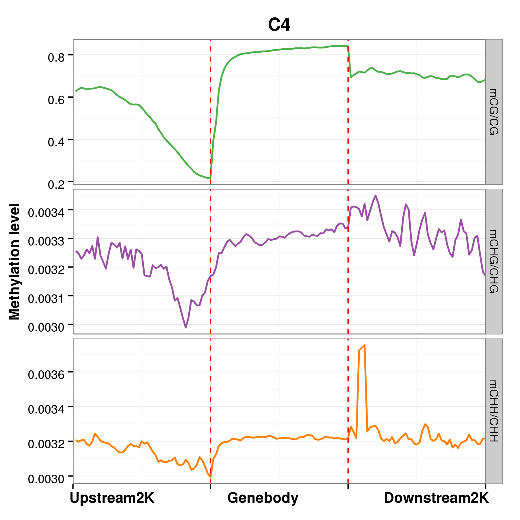 | 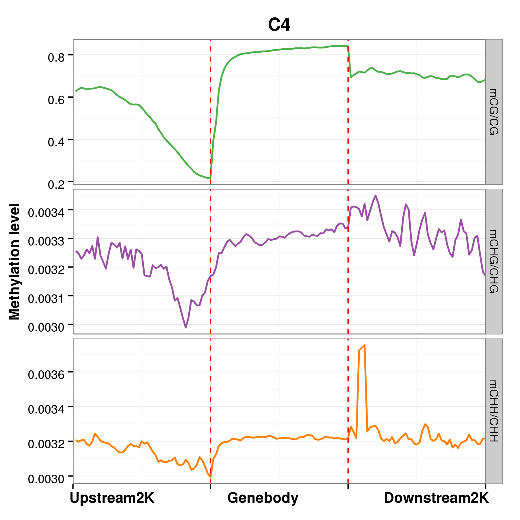 |
| 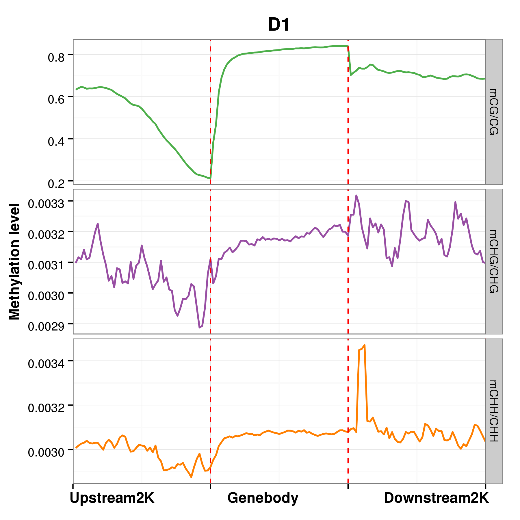 | 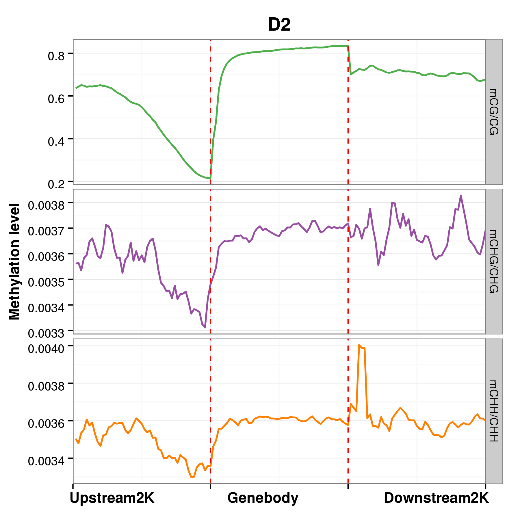 | 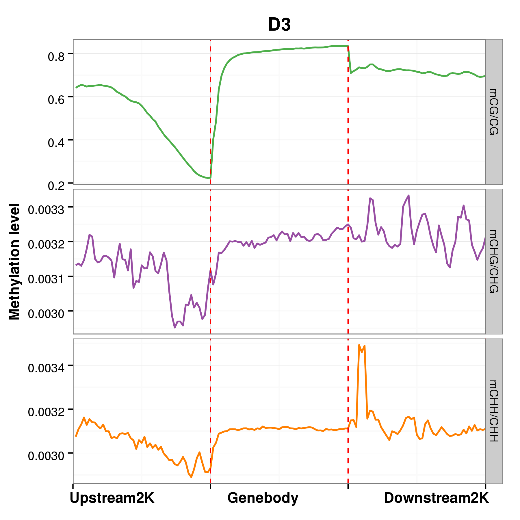 | 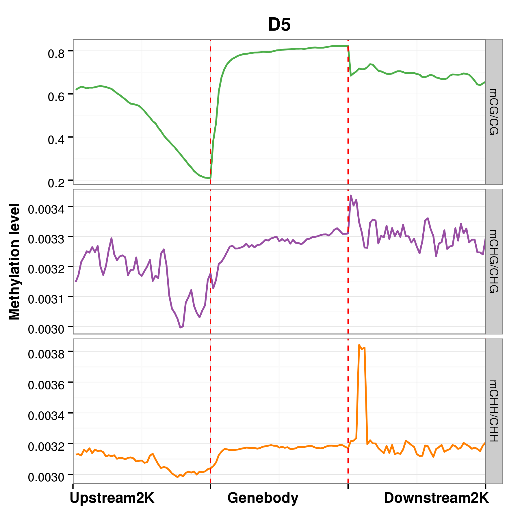 |

Supplement: Supplementary file 4 — Supplementary Material 4 (A and B)- Supplementary Figure S1 (A and B): Methylation profiles for all 16 WGBS samples across genomic features (A) and genes (B). For group information on sample ID, refer to Table S1 [file 12864_2024_10574_MOESM4_ESM.docx]

| **Group 1 v Group 2** | **Group 1 v Group 3** | **Group 1 v Group 4** |
| --- | --- | --- |
| 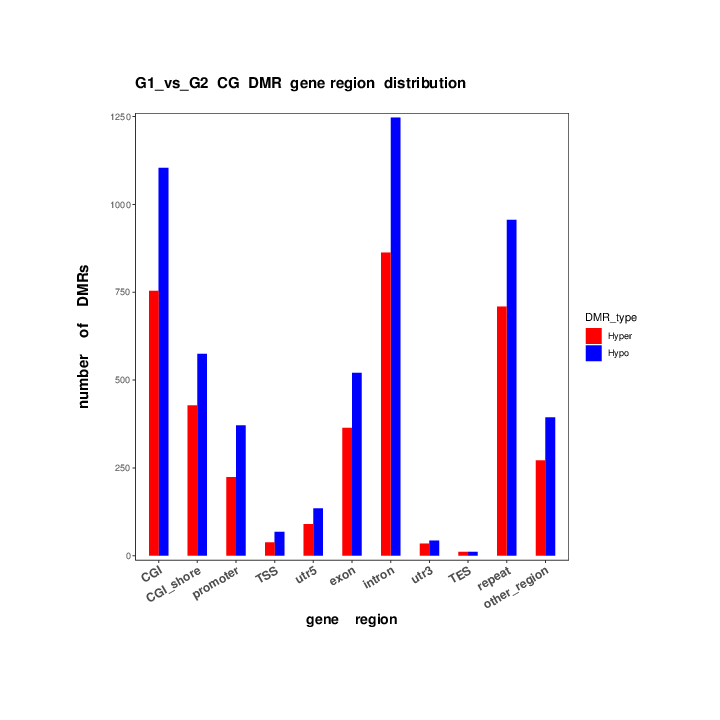 | 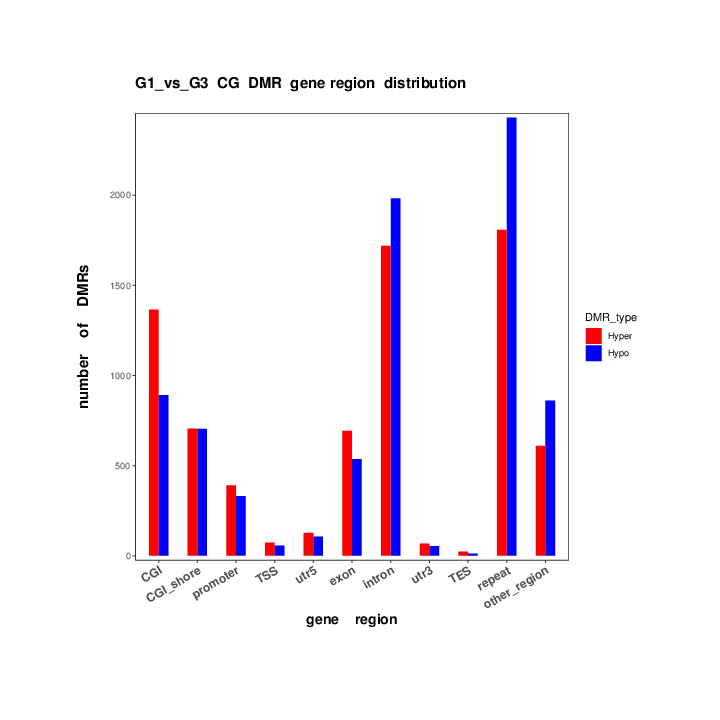 | 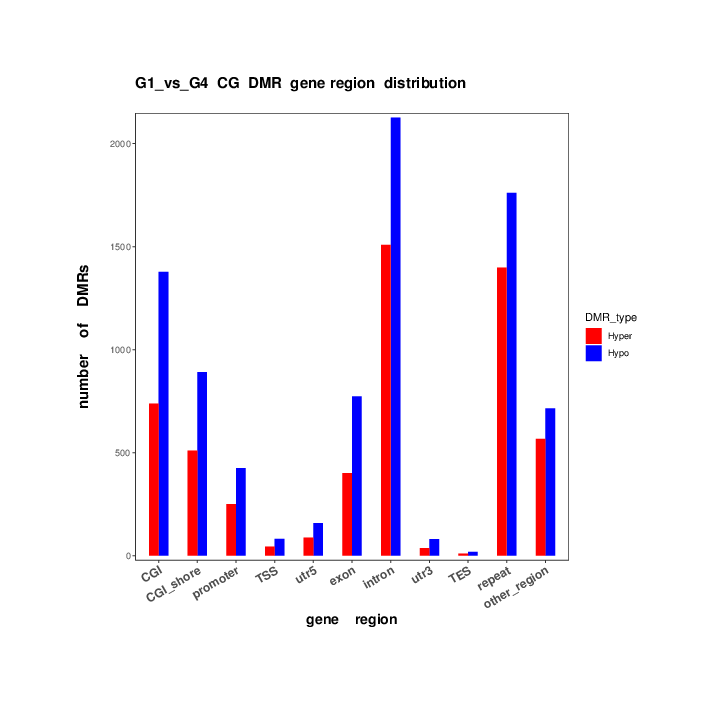 |
| 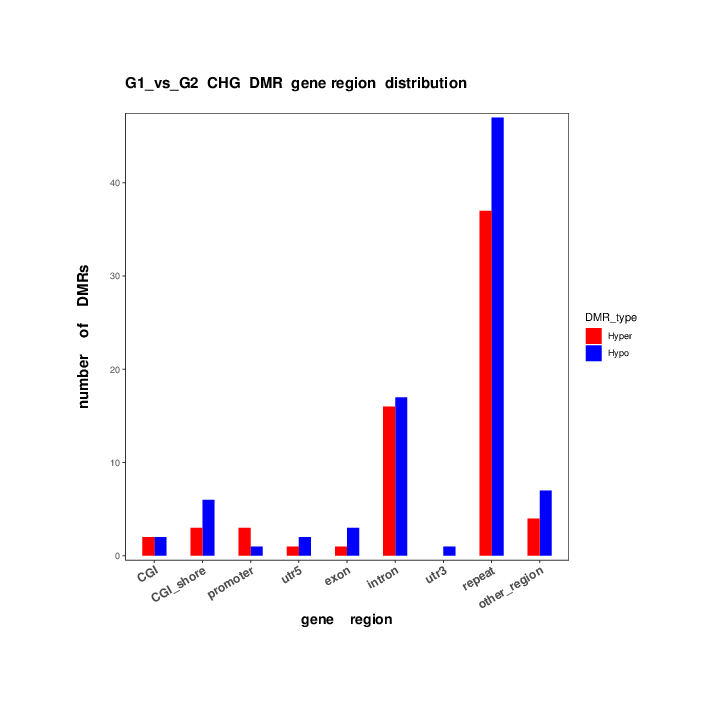 | 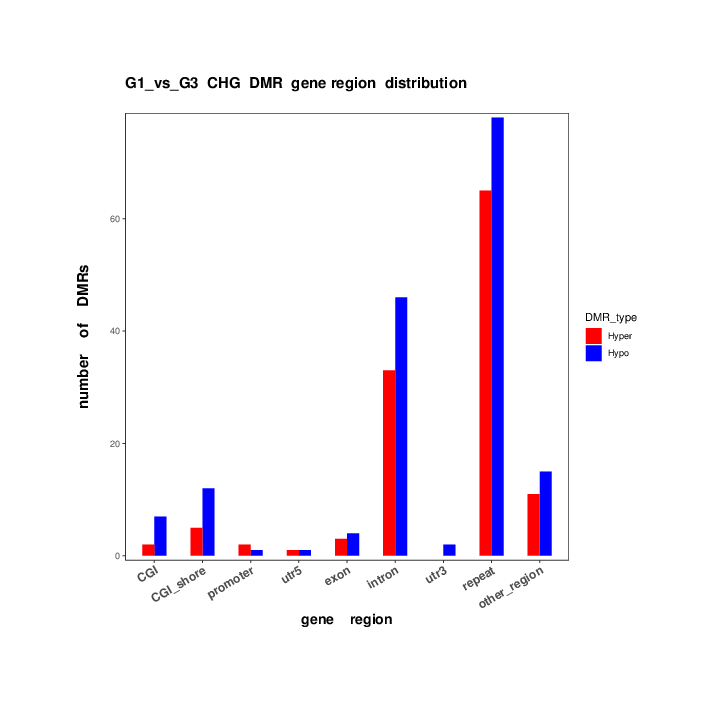 | 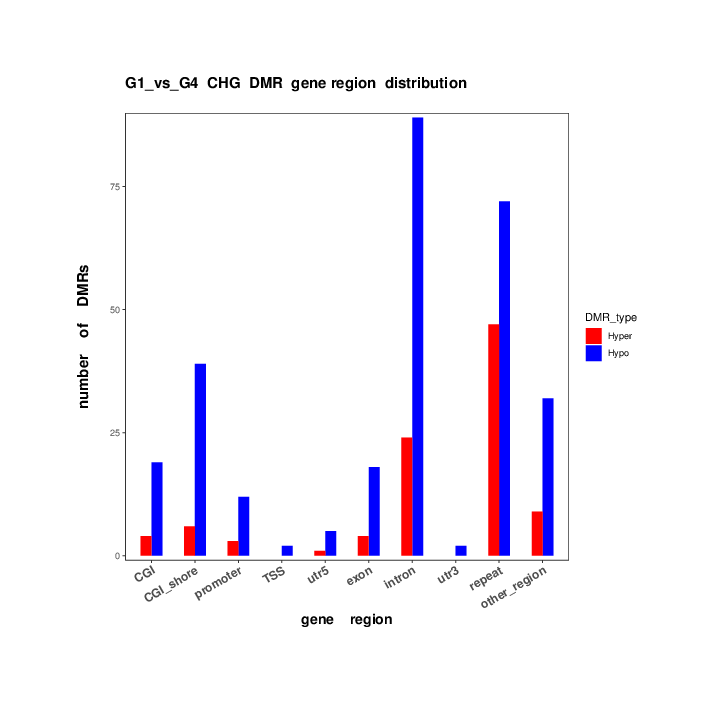 |
| 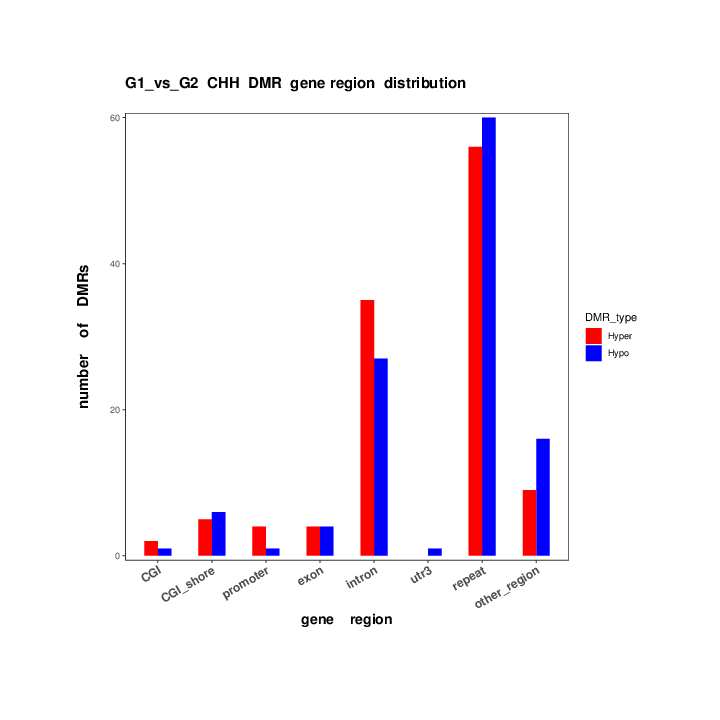 | 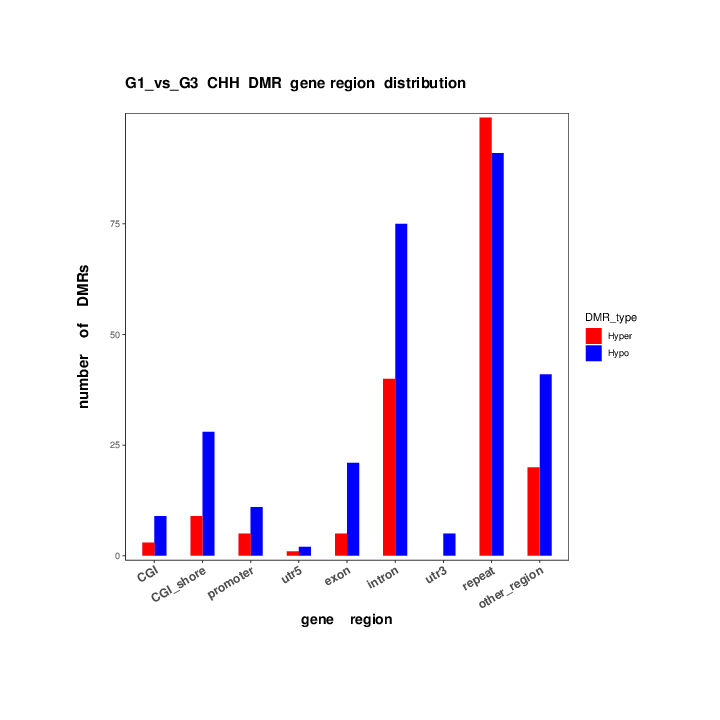 | 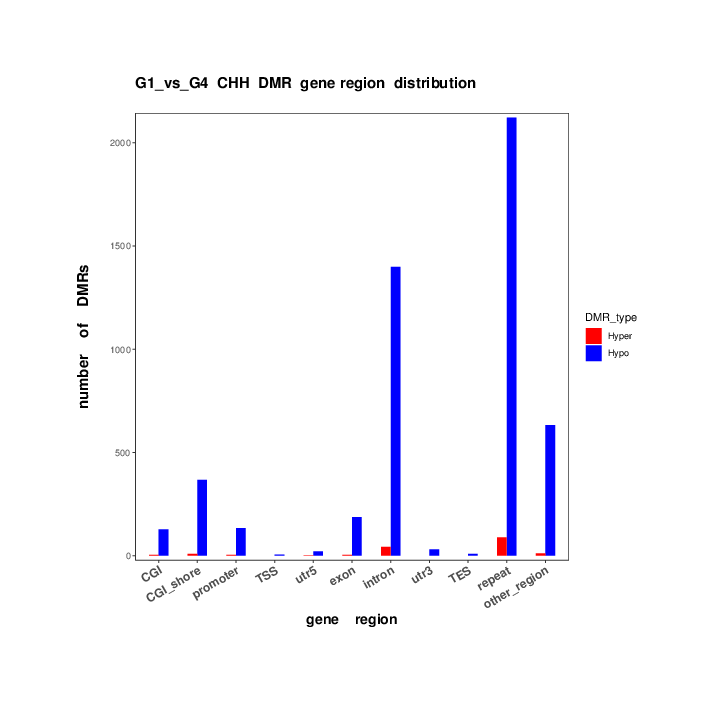 |

| **Group 2 v Group 3** | **Group 2 v Group 4** | **Group 3 v Group 4** |
| --- | --- | --- |
| 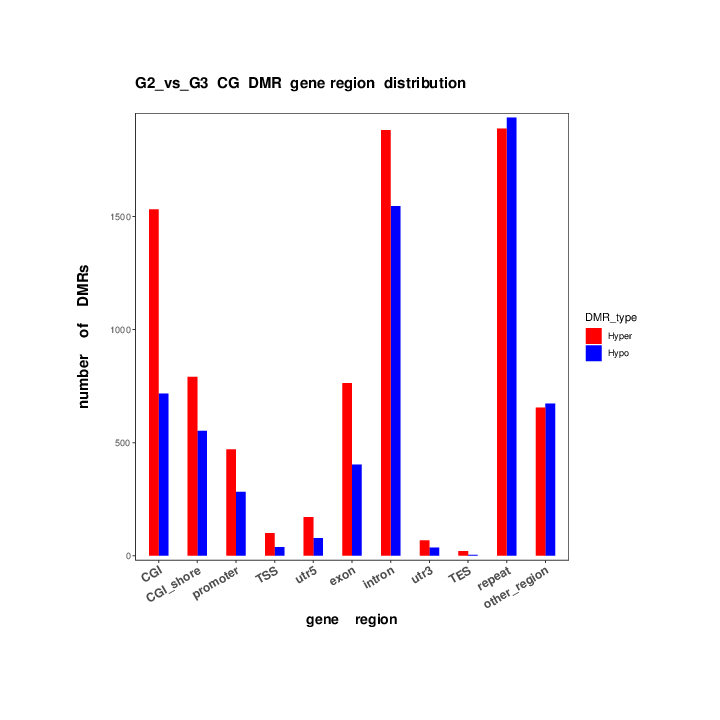 | 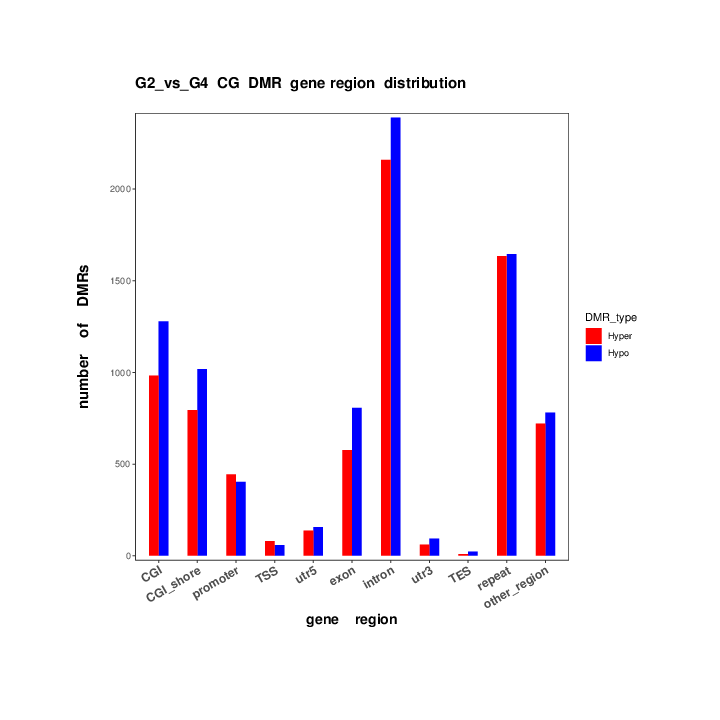 | 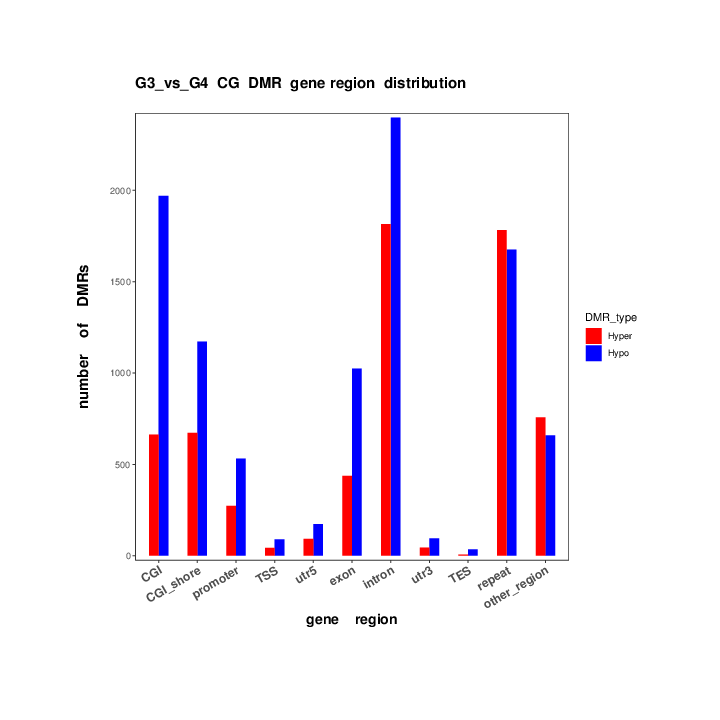 |
| 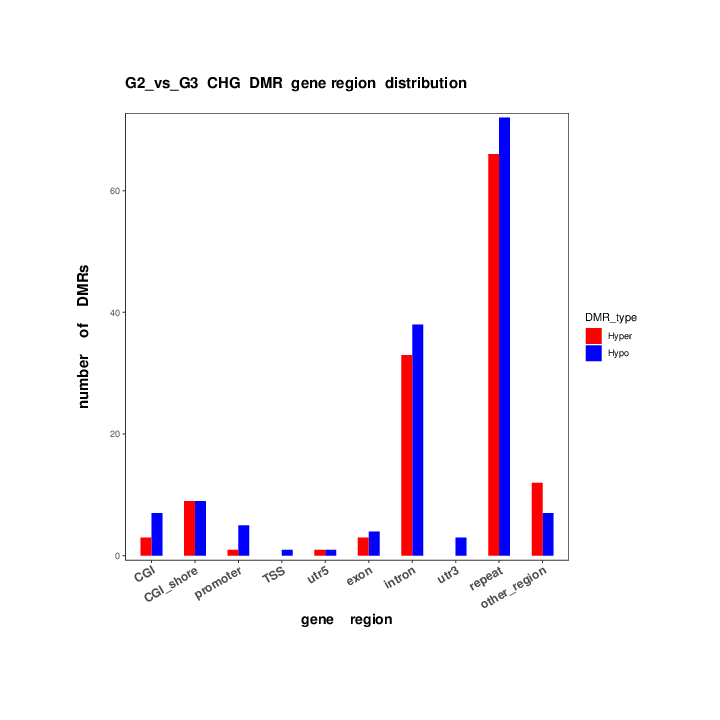 | 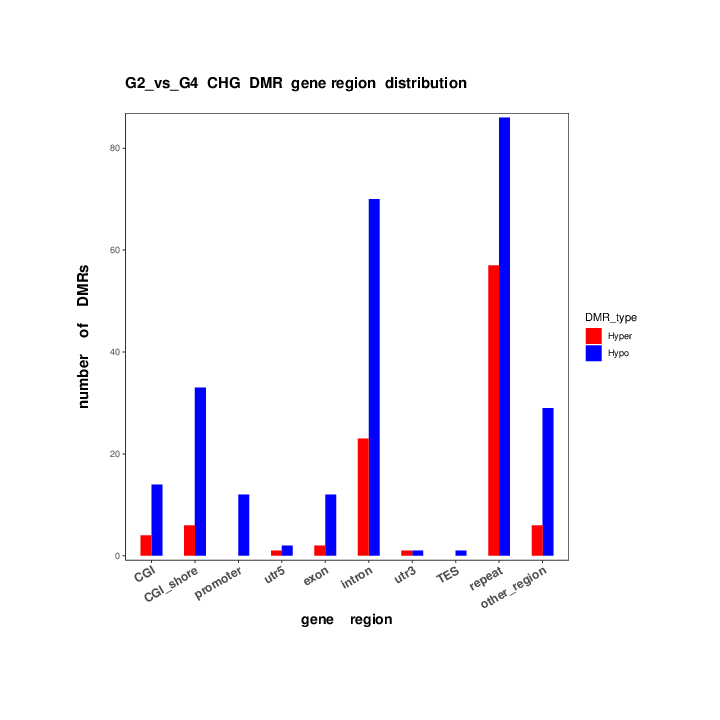 | 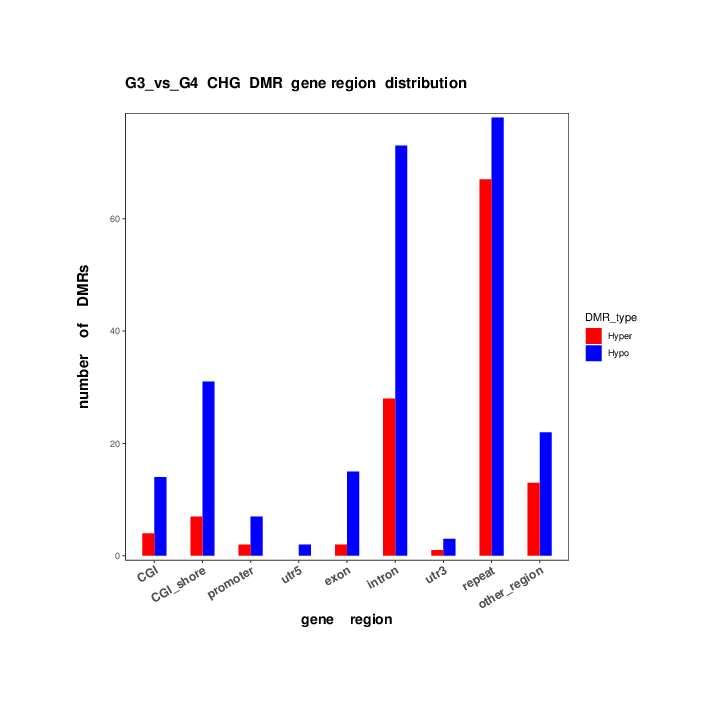 |
| 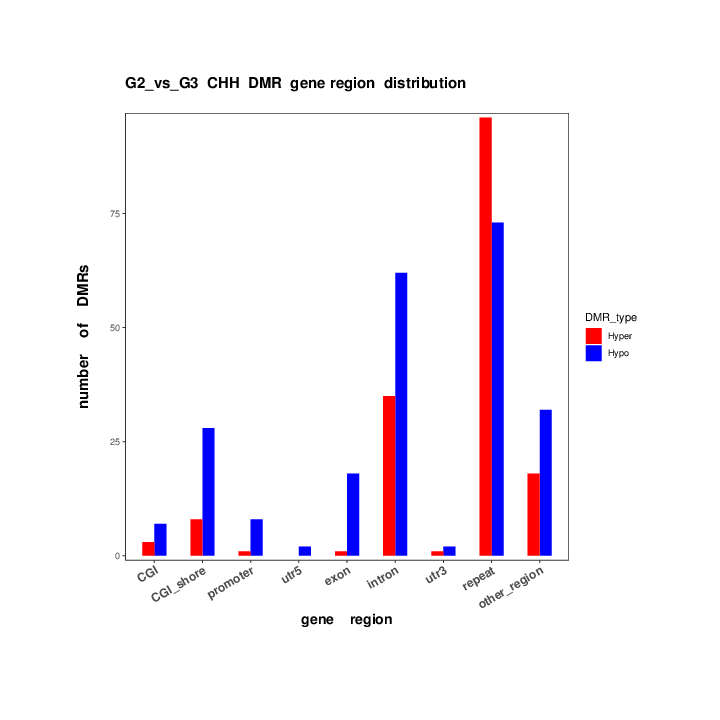 | 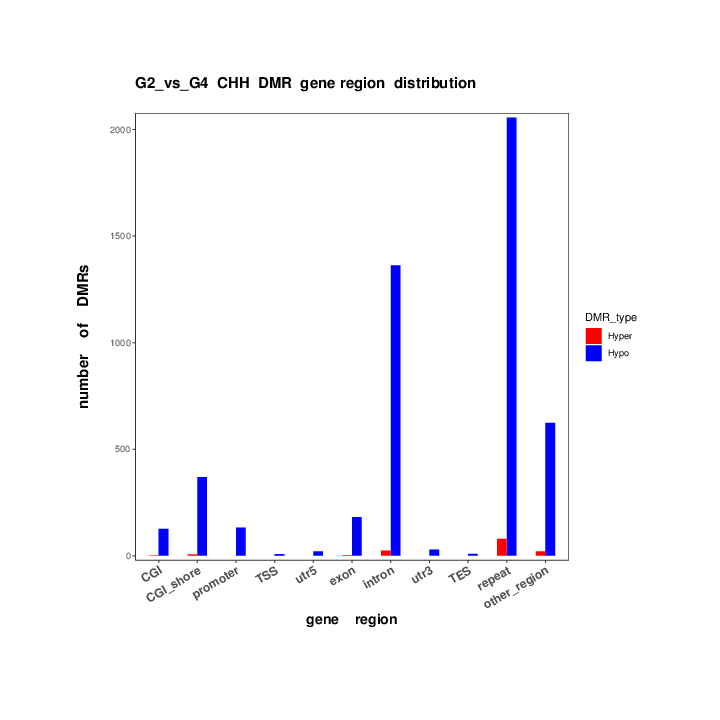 | 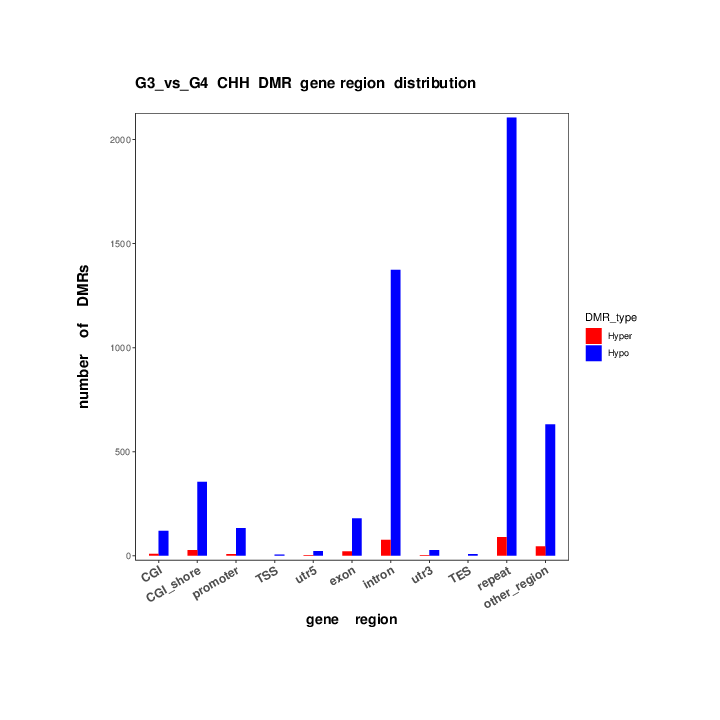 |

Supplement: Supplementary file 14 — Supplementary Material 14 - Figure S3: Ratio of hypermethylation to hypomethylation profiles for all 16 WGBS samples, divided according to experimental groups (as shown in Figure 1) across each genomic feature. Histograms show ratio for CG, CHG and CHH methylation types for each pairwise group comparison [file 12864_2024_10574_MOESM14_ESM.docx]

| **Group 1 v Group 2** | **Group 1 v Group 3** | **Group 1 v Group 4** |
| --- | --- | --- |
| 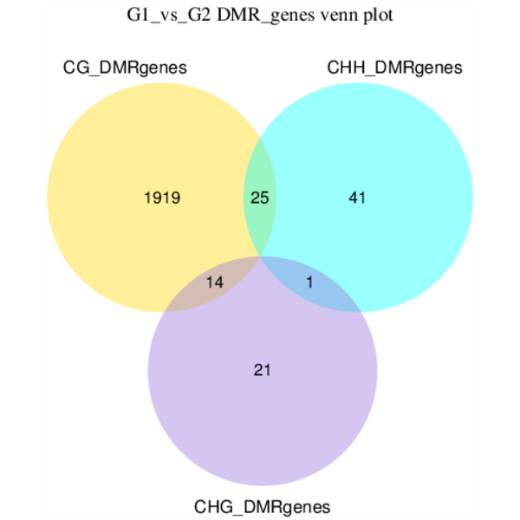 | 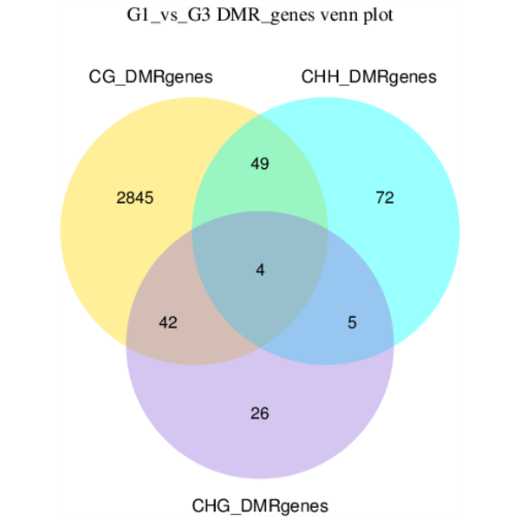 | 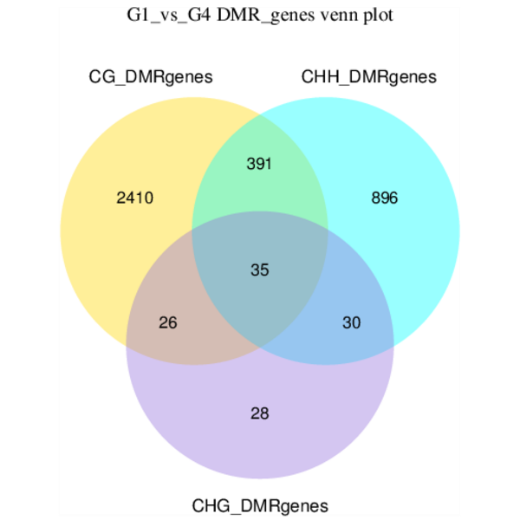 |
| 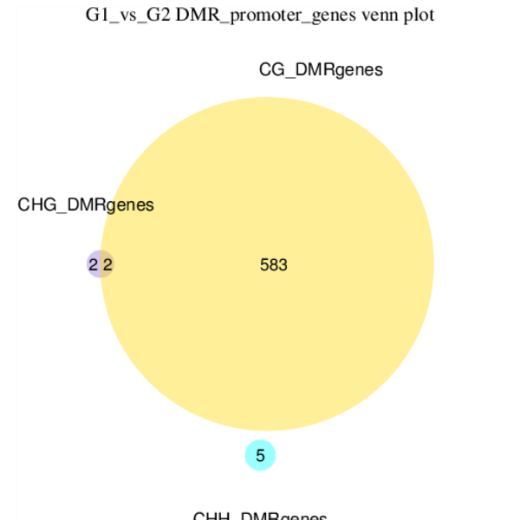 | 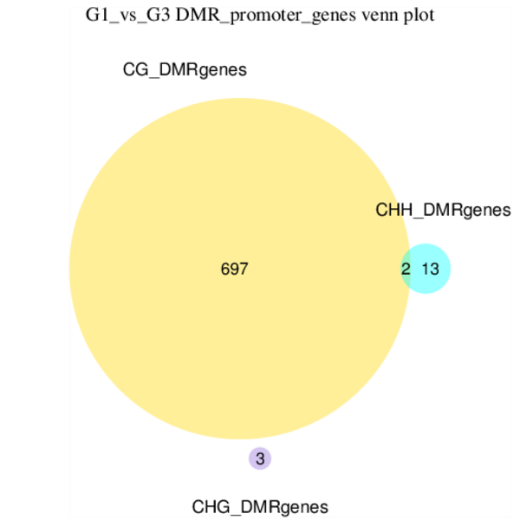 | 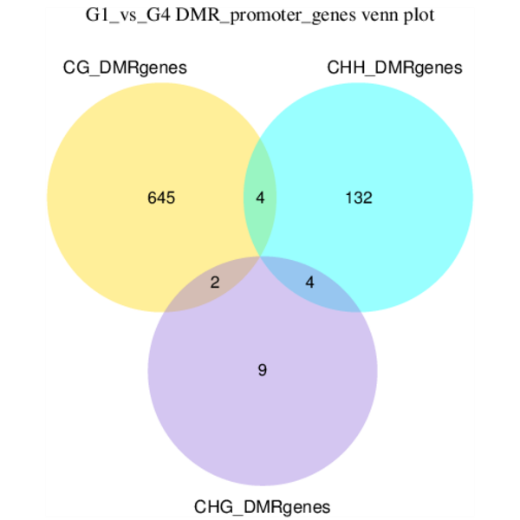 |

| **Group 2 v Group 3** | **Group 2 v Group 4** | **Group 3 v Group 4** |
| --- | --- | --- |
| 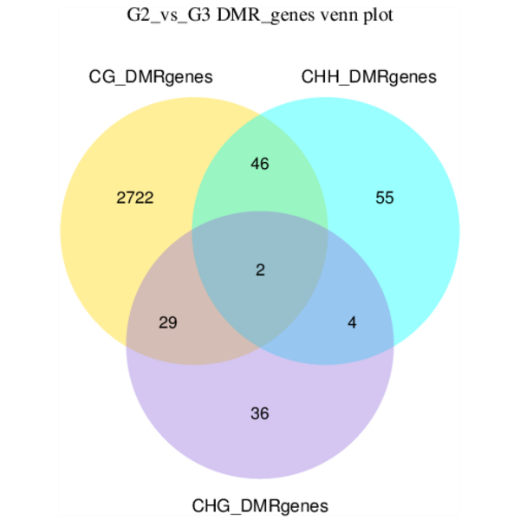 | 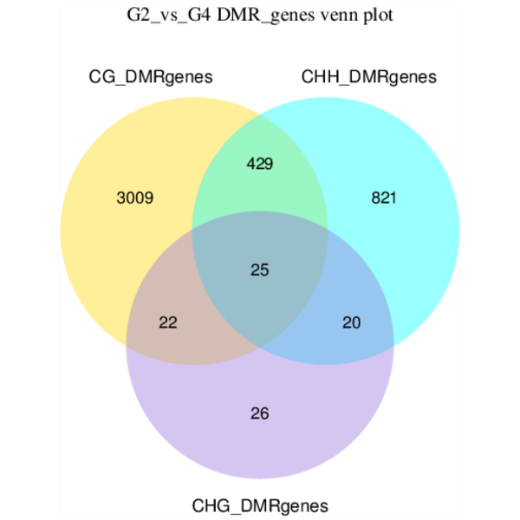 | 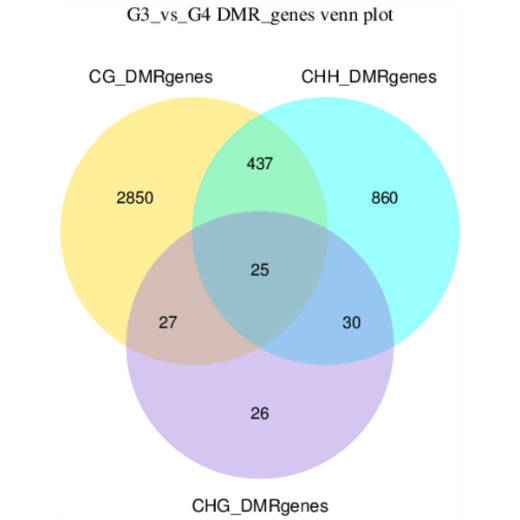 |
| 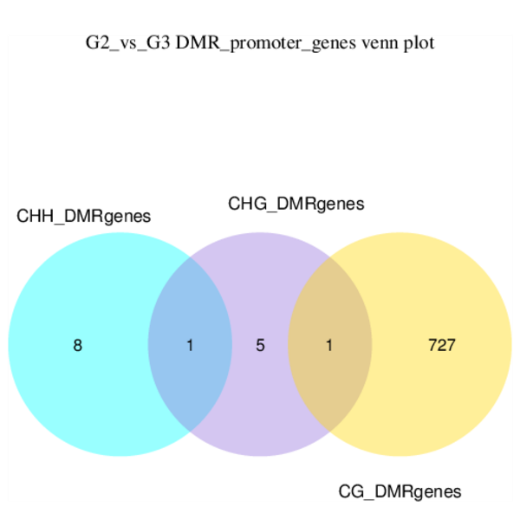 | 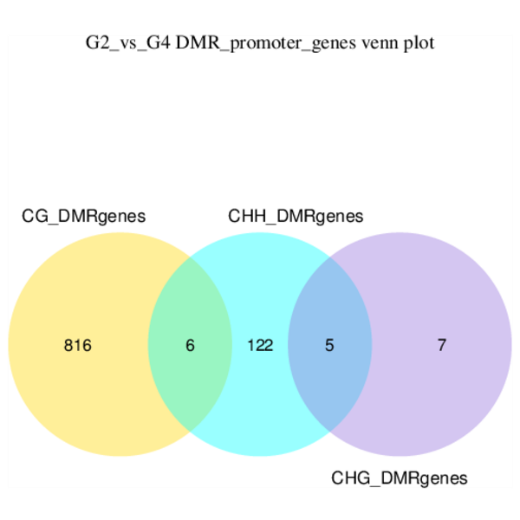 | 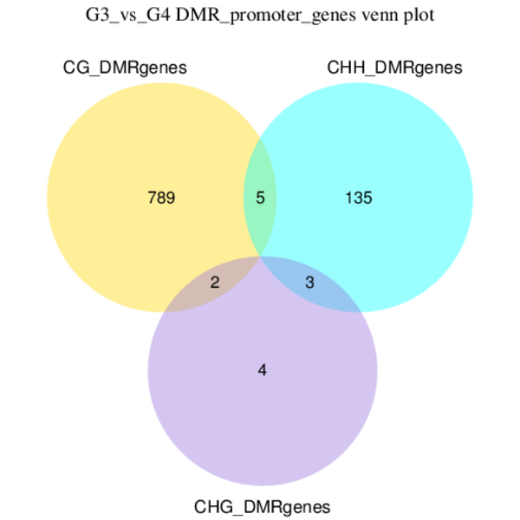 |

Supplement: Supplementary file 15 — Supplementary Material 15 - Figure S4: Numbers of genes identified as differentially methylated (CG, CHG or CHH) in DMR regions and in the promoter region of genes for all 16 WGBS samples, divided according to experimental groups (as shown in Figure 1). The degree of overlap shows the numbers of genes with multiple forms of methylation [file 12864_2024_10574_MOESM15_ESM.docx]

| **Group 1 v Group 2** | **Group 1 v Group 3** | **Group 1 v Group 4** |
| --- | --- | --- |
| 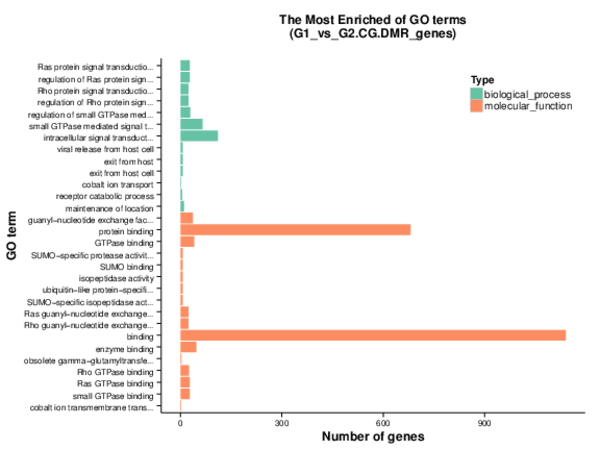 | 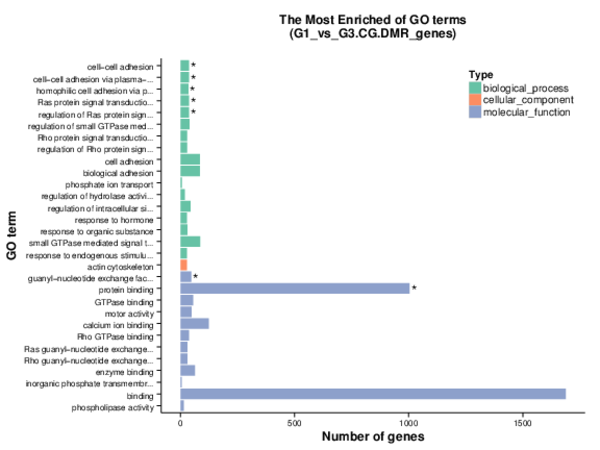 | 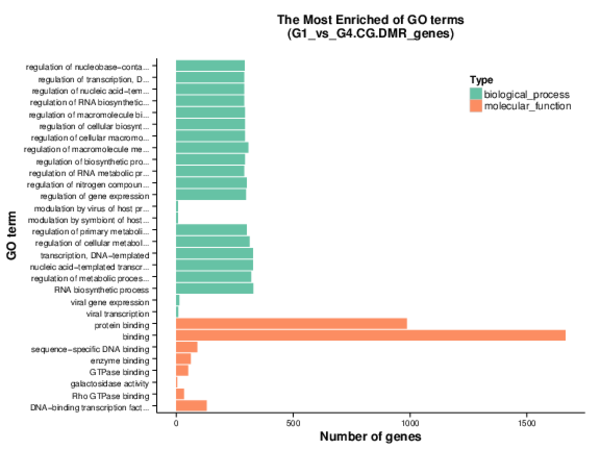 |
| 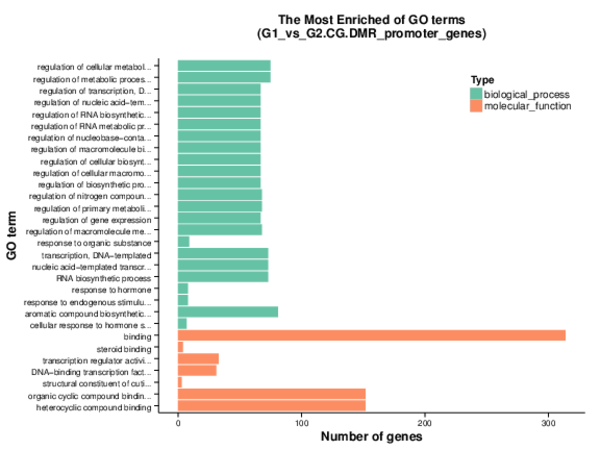 | 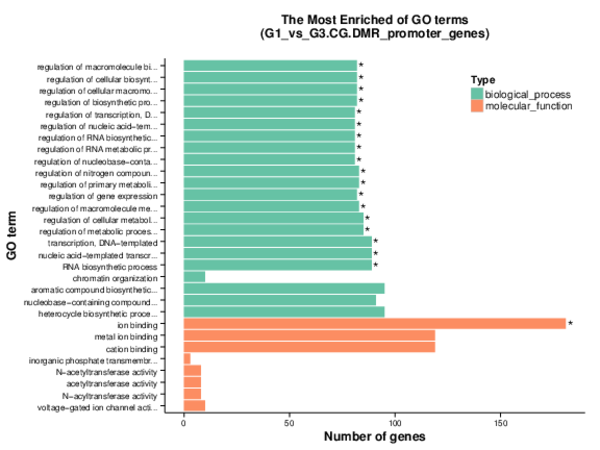 | 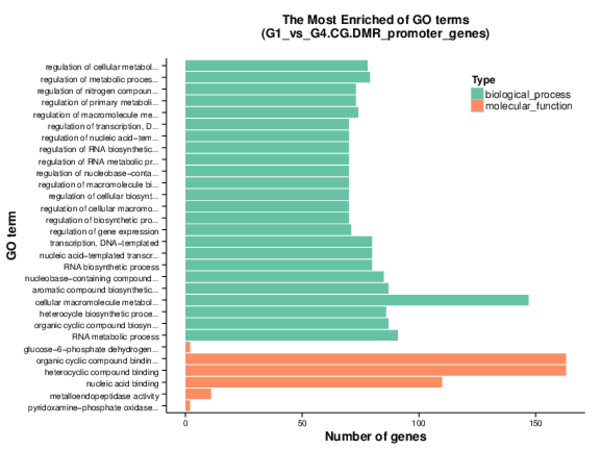 |

| **Group 2 v Group 3** | **Group 2 v Group 4** | **Group 3 v Group 4** |
| --- | --- | --- |
| 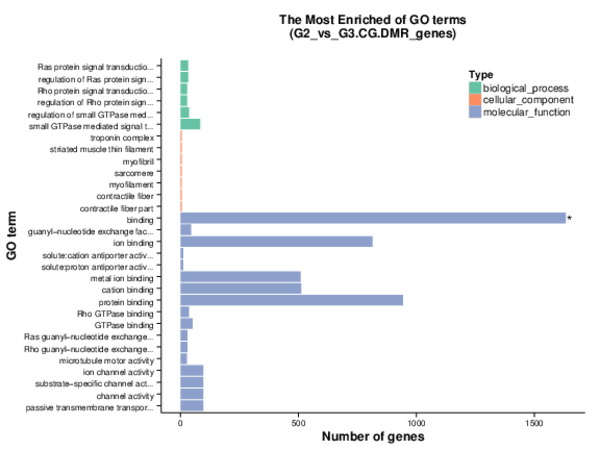 | 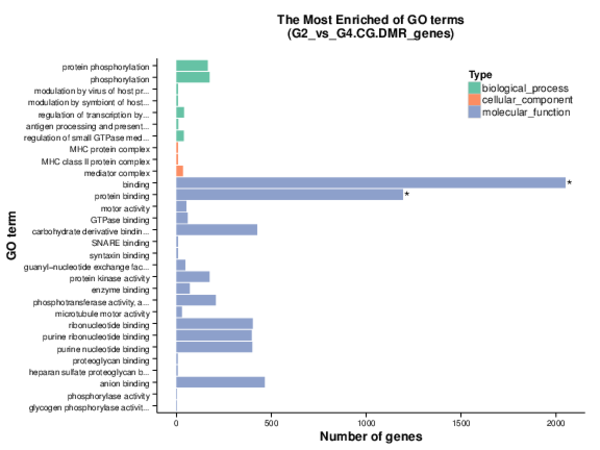 | 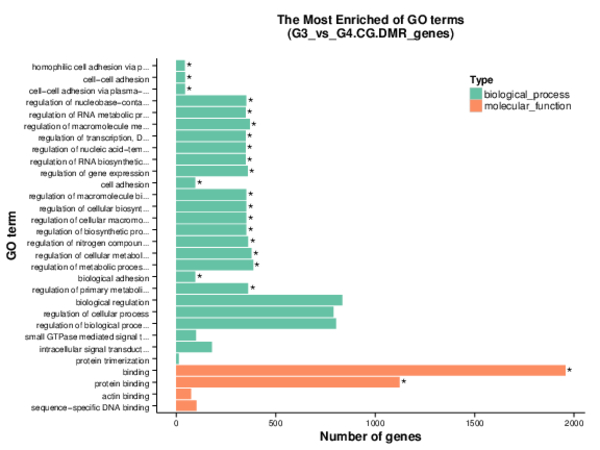 |
| 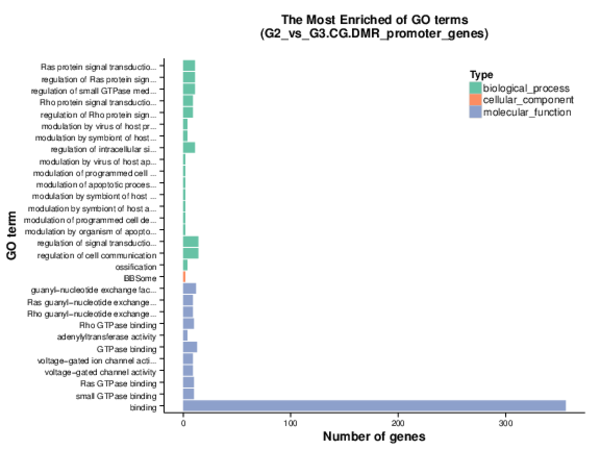 | 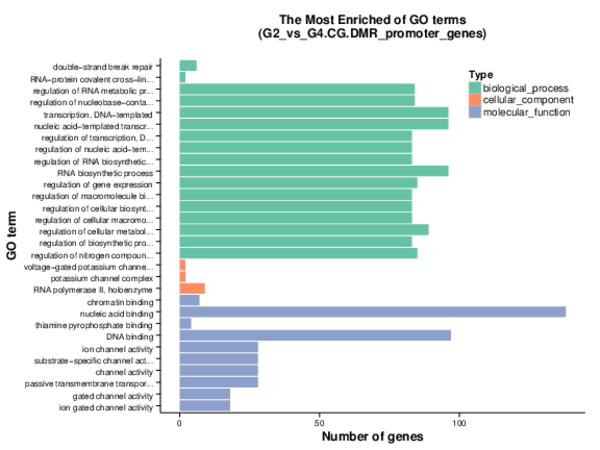 | 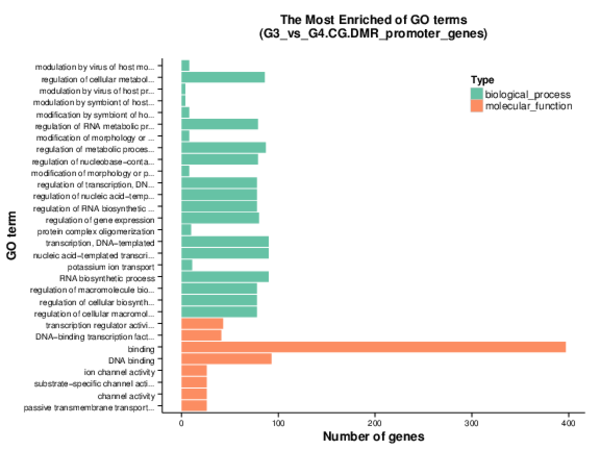 |

Supplement: Supplementary file 16 — Supplementary Material 16 - Figure S5: GO plot enrichment of biological processes, cellular component and molecular function for all 16 WGBS samples, divided according to experimental groups (as shown in Figure 1). Significantly enriched GO categories are identified with an asterix [file 12864_2024_10574_MOESM16_ESM.docx]

| **Group 1 v Group 2** | **Group 1 v Group 3** | **Group 1 v Group 4** |
| --- | --- | --- |
| 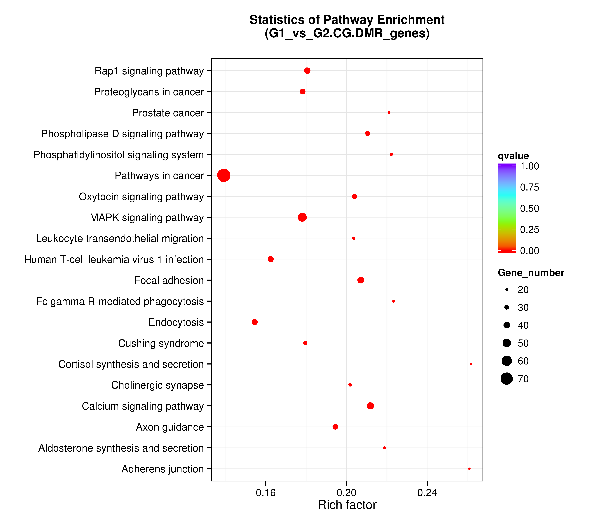 | 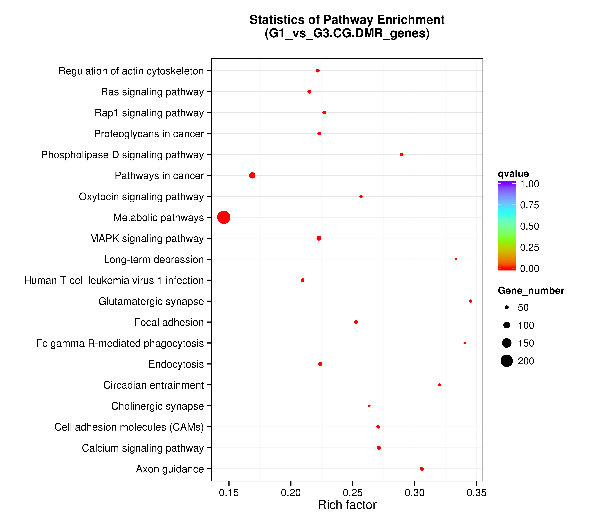 | 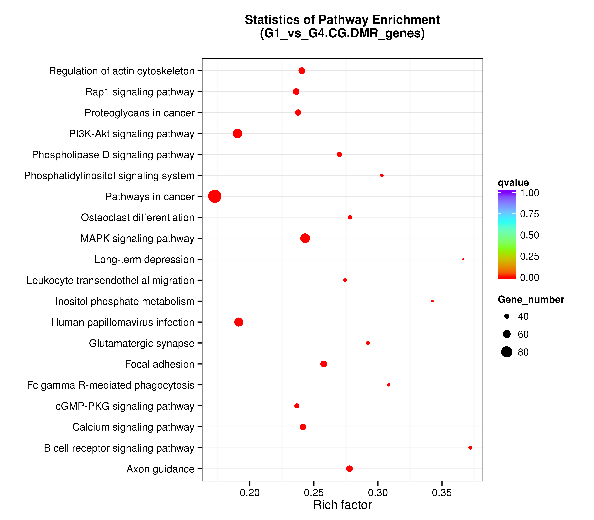 |
| 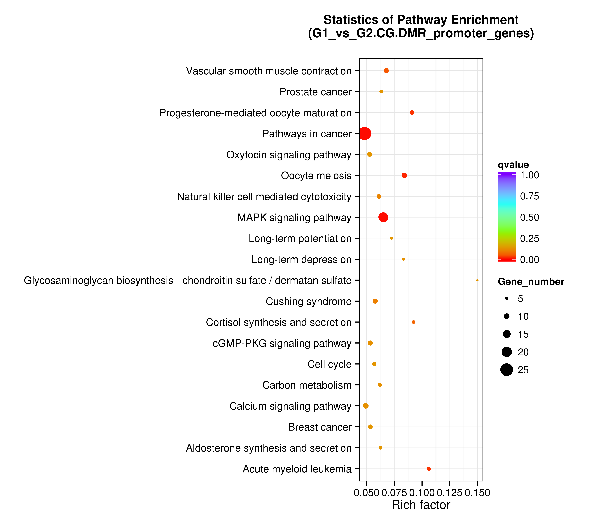 | 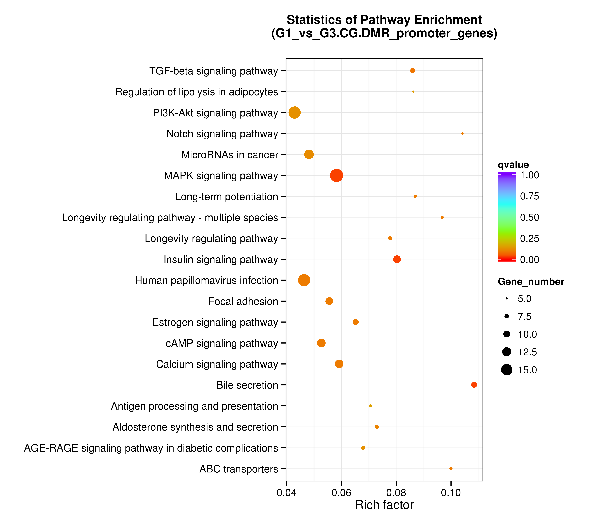 | 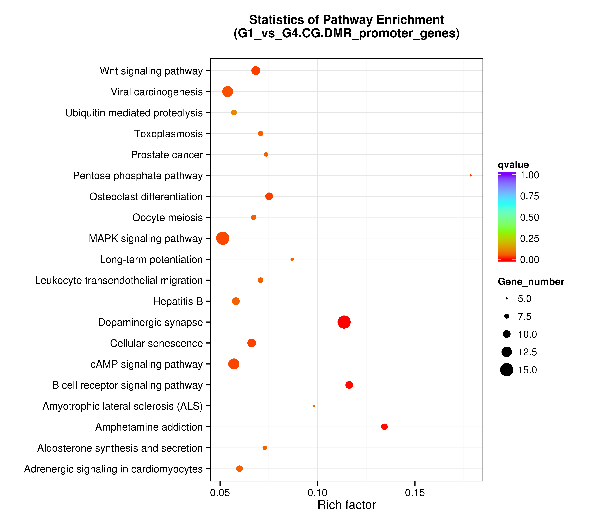 |

| **Group 2 v Group 3** | **Group 2 v Group 4** | **Group 3 v Group 4** |
| --- | --- | --- |
| 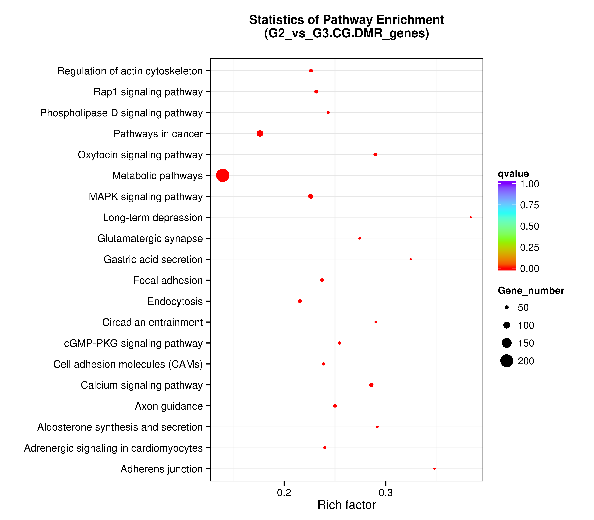 | 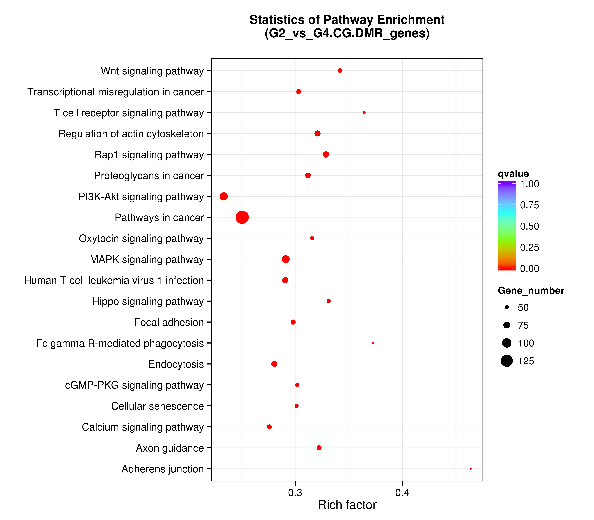 | 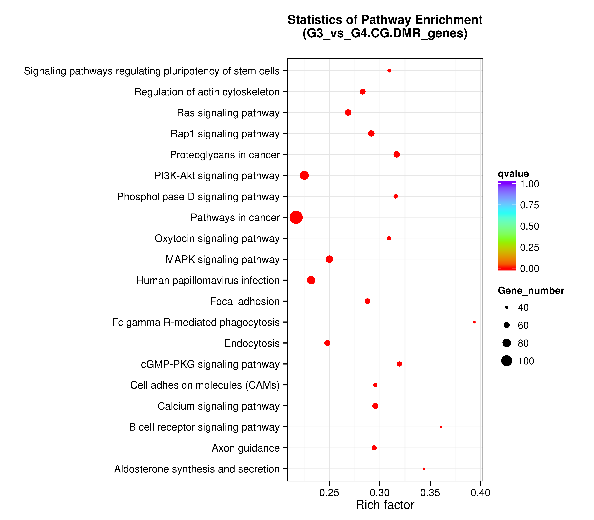 |
| 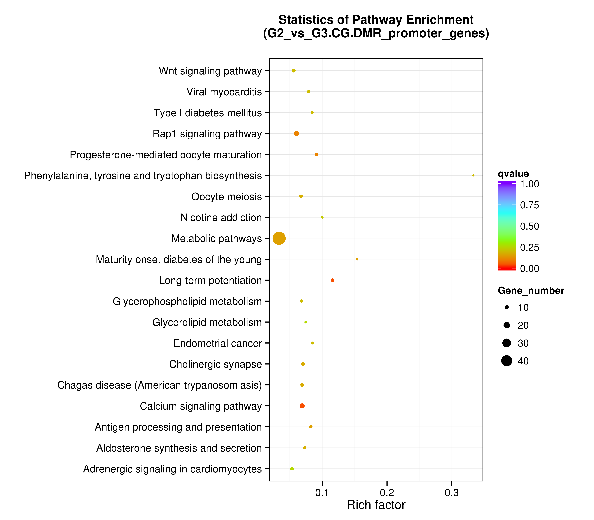 | 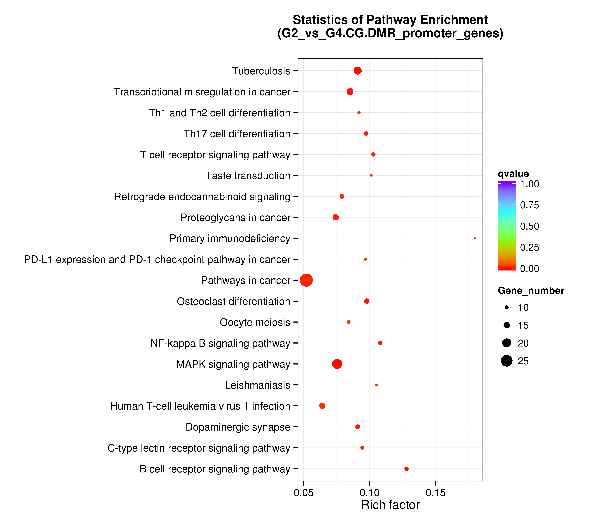 | 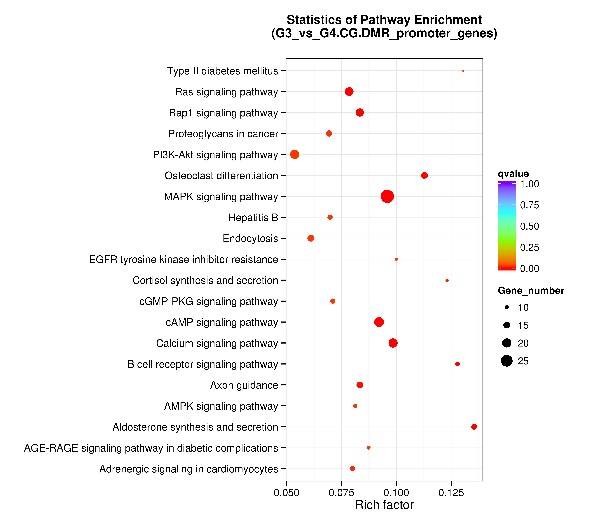 |

Supplement: Supplementary file 17 — Supplementary Material 17 - Figure S6: Scatterplot showing significantly enriched pathways from DMRs and DPMGs for all 16 WGBS samples, divided according to experimental groups (as shown in Figure 1) identified using KEGG. Corrected P values (Q value) is indicated by the colour and the numbers of genes represented in the pathway is indicated by the size of the circle. The Rich factor identifies the ratio of differentially expressed gene numbers annotated in this pathway term relative to all gene numbers annotated in this pathway term [file 12864_2024_10574_MOESM17_ESM.docx]
