# Supplementary material for: Extensive differential DNA methylation between tuberculosis skin test positive and skin test negative cattle
Source: BMC Genomics. 2024 Aug 6;25:762. doi: 10.1186/s12864-024-10574-x (PMC11301934; doi:10.1186/s12864-024-10574-x)
Supplement: Supplementary file 13 — Supplementary Material 13 - Figure S2: Differential methylation profiles for all 16 WGBS samples, divided according to experimental groups (as shown in Figure 1). Methylation plots are shown for combined levels of CG, CHG and CHH methylation types as well as each type individually for pairwise each group comparison [file 12864_2024_10574_MOESM13_ESM.docx]

| **M** | **Group 1 v Group 2** | **Group 1 v Group 3** | **Group 1 v Group 4** |
| --- | --- | --- | --- |
| Region all | 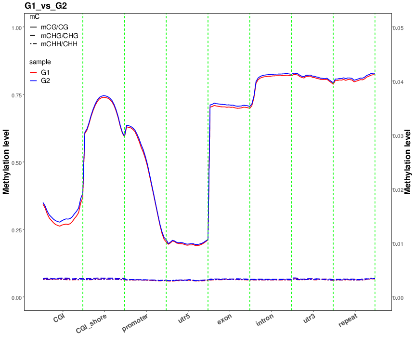 | 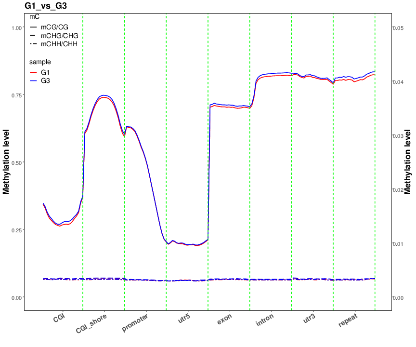 | 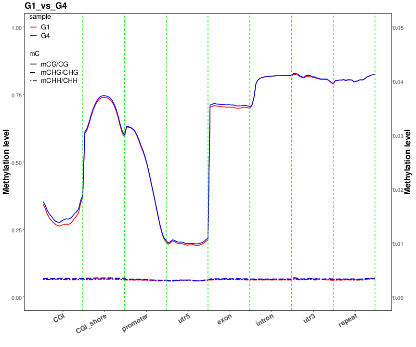 |
| mCG | 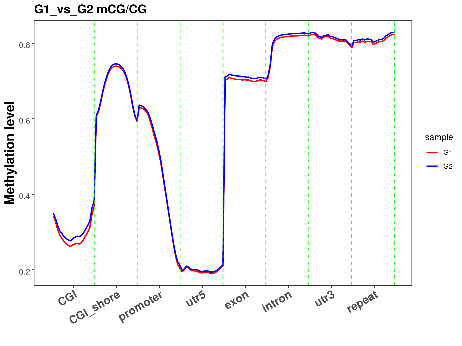 | 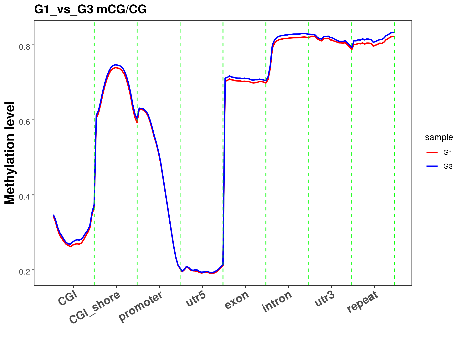 | 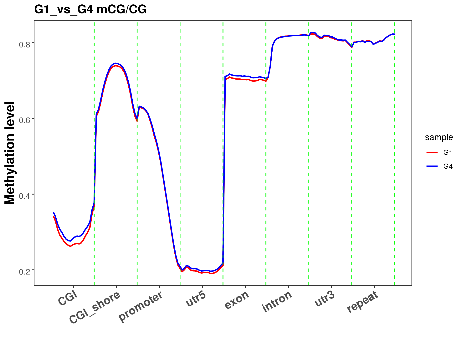 |
| mCHG | 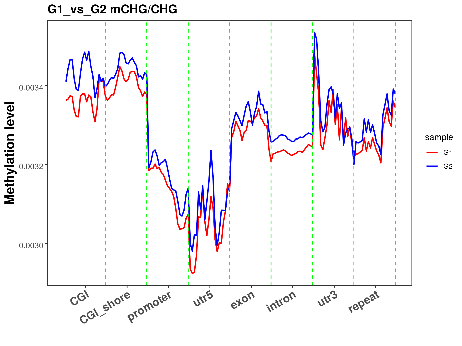 | 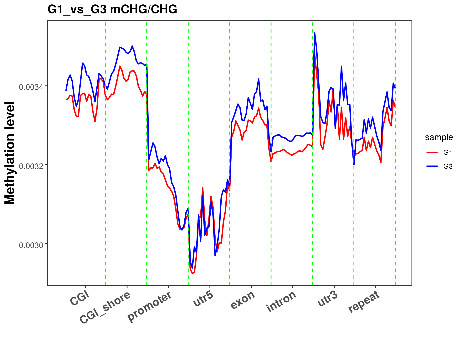 | 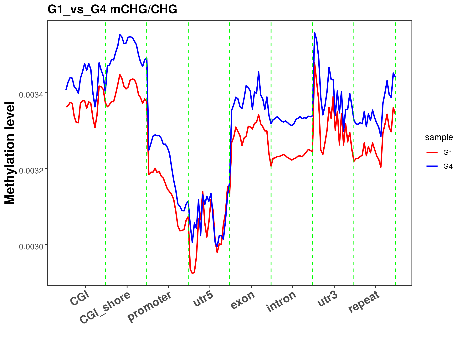 |
| mCHH | 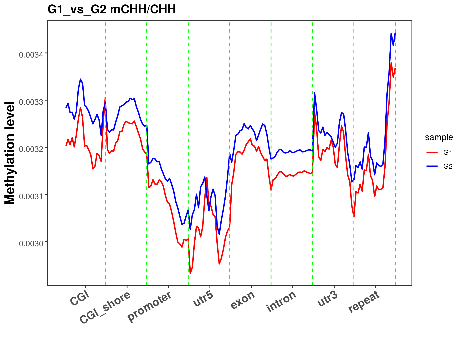 | 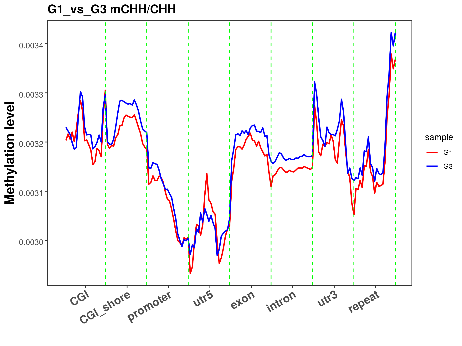 | 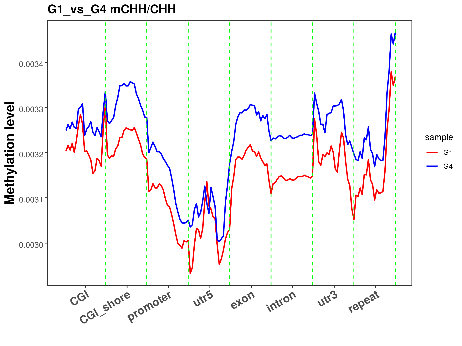 |

| **M** | **Group 2 v Group 3** | **Group 2 v Group 4** | **Group 3 v Group 4** |
| --- | --- | --- | --- |
| Region all | 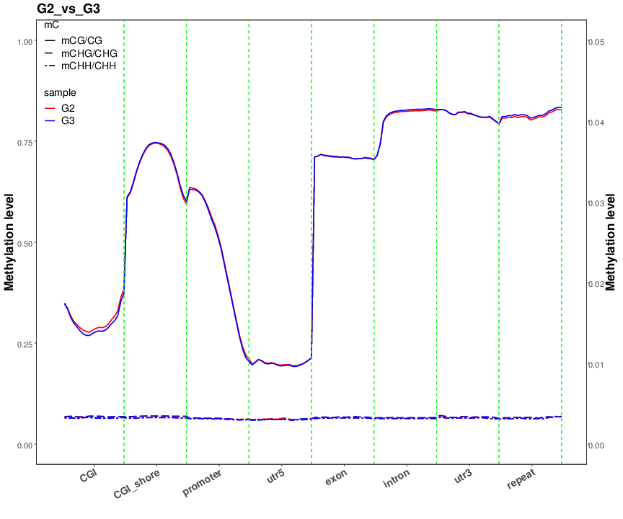 | 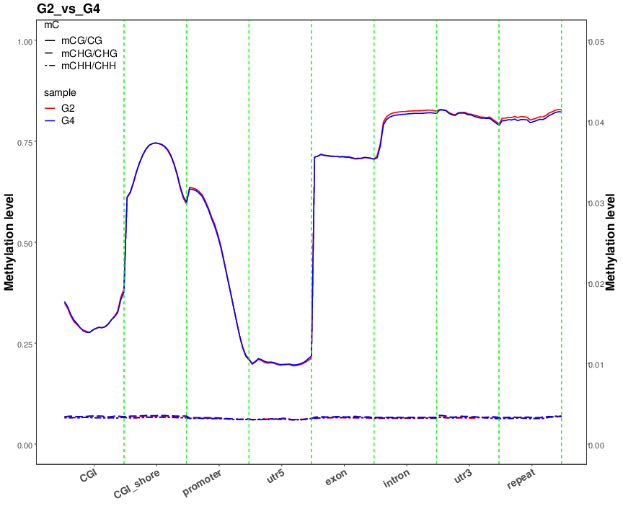 | 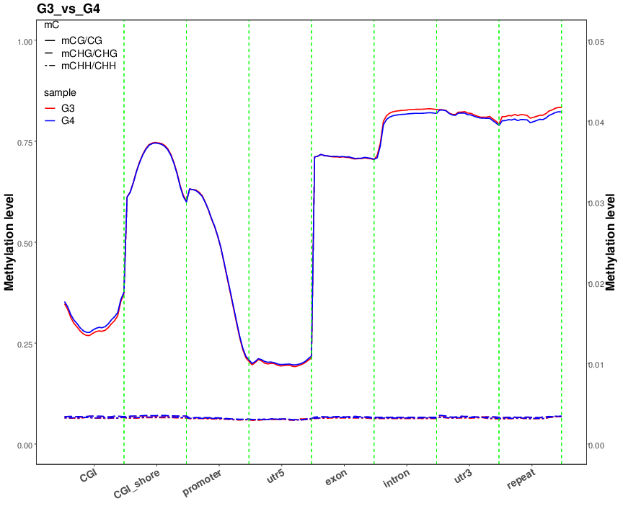 |
| mCG | 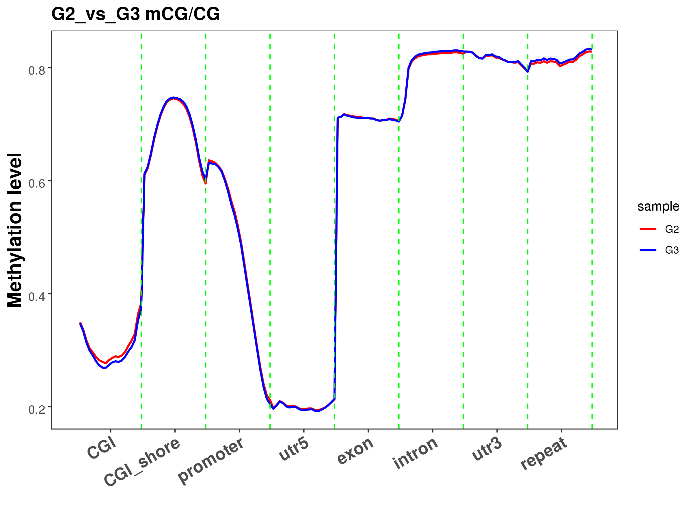 | 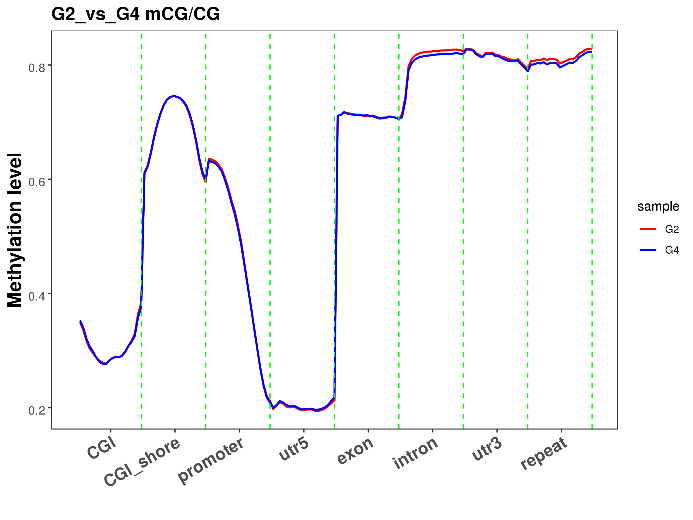 | 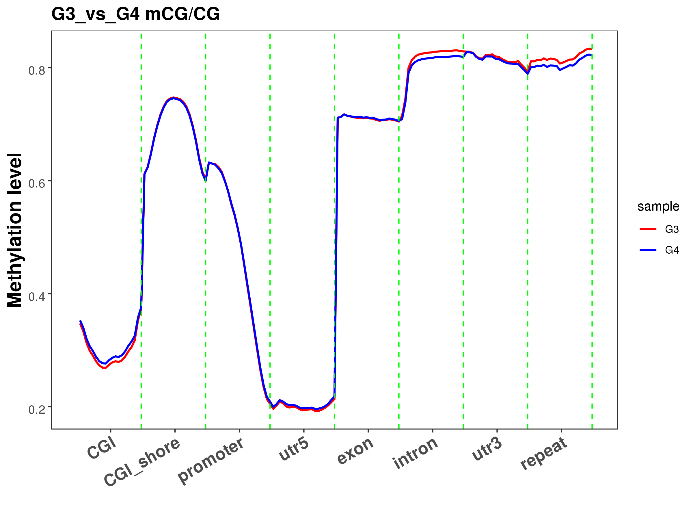 |
| mCHG | 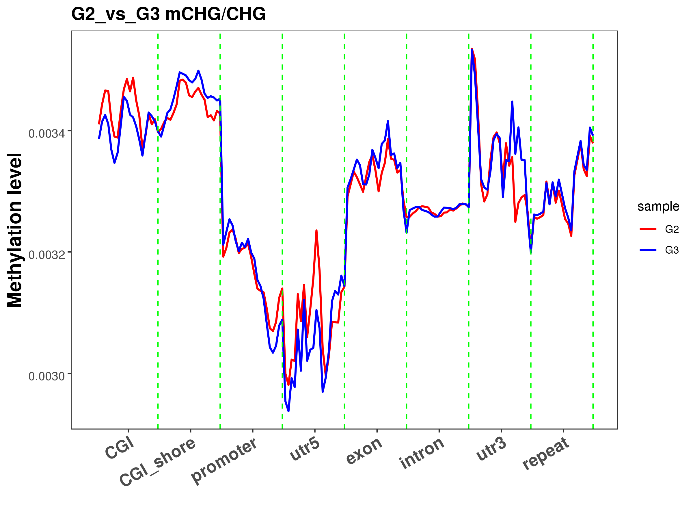 | 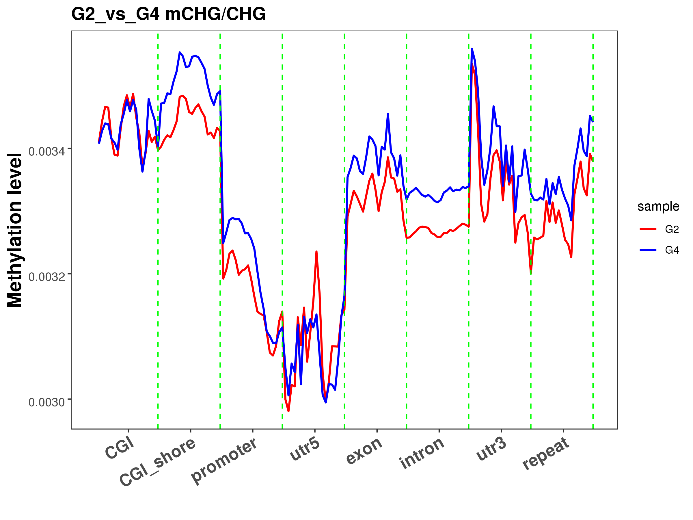 | 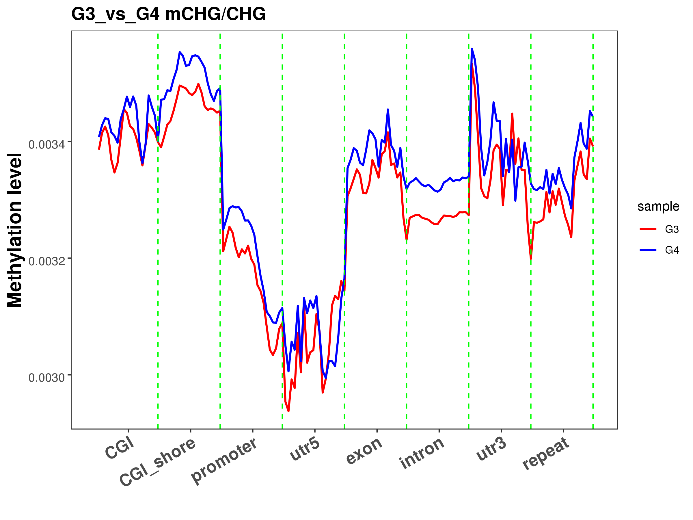 |
| mCHH | 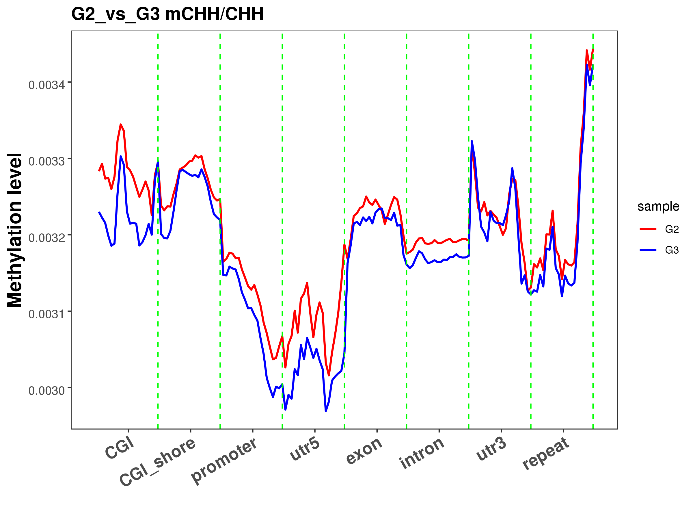 | 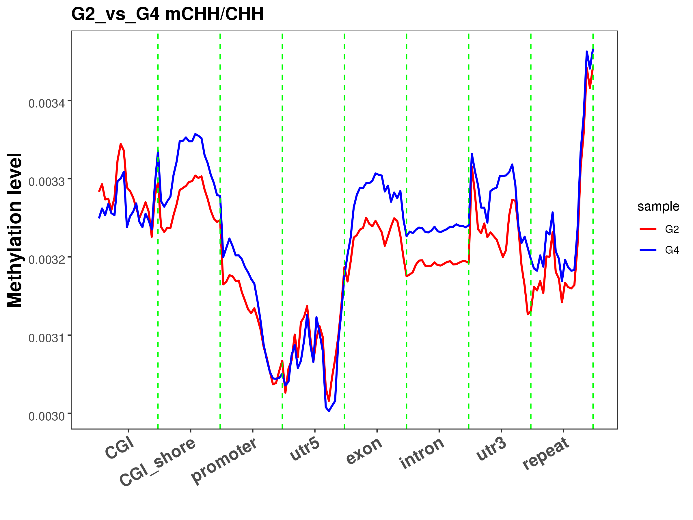 | 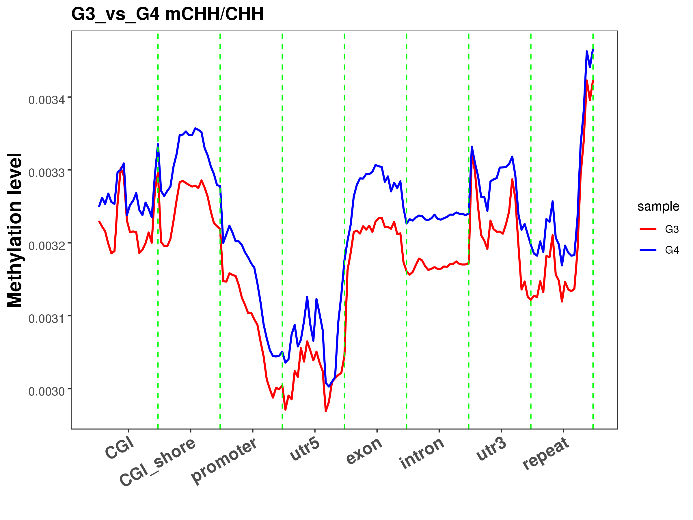 |
